# Long-Term Outcomes after Use of Perioperative Glucocorticoids in Patients Undergoing Cancer Surgery: A Systematic Review and Meta-Analysis

**DOI:** 10.3390/cancers12010076

**Published:** 2019-12-27

**Authors:** Emma Rosenkrantz Hölmich, Rune Petring Hasselager, Michael Tvilling Madsen, Adile Orhan, Ismail Gögenur

**Affiliations:** 1 Center for Surgical Science, Department of Surgery, Zealand University Hospital, Lykkebækvej 1, 4600 Køge, Denmark; rubh@regionsjaelland.dk (R.P.H.); mitm@regionsjaelland.dk (M.T.M.); aor@regionsjaelland.dk (A.O.); igo@regionsjaelland.dk (I.G.); 2 Department of Clinical Medicine, University of Copenhagen, Blegdamsvej 3B, 2200 Copenhagen N, Denmark

**Keywords:** oncoanaesthesiology, surgery, anesthesiology, cancer, perioperative treatment, steroids, corticosteroids, glucocorticoids, recurrence, survival

## Abstract

The surgical stress response can accelerate clinical metastasis formation. Perioperative glucocorticoids might modulate this response and the metastatic process. We aimed to describe associations between perioperative glucocorticoids and long-term outcomes after cancer surgery. We searched four databases for eligible trials and performed meta-analyses on frequency and time-to-event data. We included sixteen studies that evaluated eight different cancer types. No association was found between perioperative glucocorticoids and recurrence in either the frequency meta-analysis, risk ratio (RR) 1.04, 95% confidence interval (CI) (0.87–1.25), or in the time-to-event meta-analysis, hazard ratio (HR) 1.18, 95% CI (0.78–1.79). Increased 1-year overall survival, RR 0.70, 95% (0.51–0.97), and disease-free survival, RR 0.77, 95% CI (0.60–0.97), was found for the glucocorticoid group, but five years after surgery, overall survival was reduced for the glucocorticoid group, RR 1.64, 95% CI (1.00–2.71). An exploratory subgroup analysis revealed decreased overall survival, HR 1.78, 95% CI (1.57–2.03), for patients undergoing colorectal cancer surgery while receiving glucocorticoids. Perioperative glucocorticoids were not associated with recurrence after cancer surgery. We found neither beneficial or deleterious associations between glucocorticoids and overall survival or disease-free survival. The available evidence remains heterogenous; low in quality and amount; and cancer-specific at present.

## 1. Introduction

The global disease burden of cancer is considerable with as much as 18 million new cases annually, and more than half as many mortalities in 2018. The majority of cancer deaths are caused by metastases [1], and the numbers are expected to nearly double in 2040 [2].

Surgical resection remains the primary curative treatment of solid cancers. This intervention induces a stress response that can contribute to the formation of clinical metastases, mainly by changes in immunological function and tumour microenvironment [3,4]. The release of prostaglandins and catecholamines leads to immune suppression and release of prometastatic factors, a combination that is essential for the high impact of the perioperative period in determining oncological outcomes [5]. Because inflammatory processes are associated with tumour growth, the surgically induced metastatic spread could possibly be altered by using perioperative immunomodulatory drugs such as glucocorticoids. Preliminary animal studies have investigated the effect of steroids on cancer progression and the results are not conclusive [6,7]. Some suggest that endogenous glucocorticoids released from prolonged stress response may hinder natural killer (NK) cell activity, which is essential in malignant defence, but the effect might not be as significant as the NK cell suppression mediated by stress related catecholamines [8]. On the other hand, the anti-inflammatory effects of glucocorticoids might prove beneficial in breaking the inflammatory cycle.

Glucocorticoids are known to modulate the inflammatory stress response and are increasingly used in the perioperative period. Administration of low-dose perioperative dexamethasone is used for prevention of postoperative nausea and vomiting (PONV). Use of high dose glucocorticoids has been shown to have analgesic effects and may even decrease the risk of postoperative complications [9] without increasing short-term adverse events [10]. It appears that glucocorticoids are safe and beneficial for immediate postoperative outcomes. However, evaluation of safety and possible benefits on long-term oncologic outcomes remain to be shown. Furthermore, it has been recommended that the effect of adjunct medications for anaesthesia on oncological outcomes should be prioritized by researchers [11].

In this study, we hypothesized that the use of perioperative glucocorticoids may impact long-term survival after cancer surgery. We aimed to describe the possible association between perioperative glucocorticoids and long-term outcomes for patients undergoing cancer surgery. The primary outcome of interest was recurrence, and the secondary outcomes were overall survival (OS); disease-free survival (DFS); and cancer-specific survival (CSS).

## 2. Materials and Methods

### 2.1. Protocol and Registration

This systematic review and meta-analysis was reported according to the PRISMA guidelines [12]. The protocol was prepared according to PRISMA-P guidelines [13] and was registered at the PROSPERO database with ID CRD42019132638 before initiation of data extraction.

### 2.2. Eligibility Criteria

The review design was built upon the population, intervention, control, outcome (PICO) framework; the population of interest being humans at 18 years or older undergoing elective surgical treatment for cancer of any kind. The intervention of interest was administration of single or repetitive doses of glucocorticoids during the perioperative period which was defined as the time ranging from one week prior to surgery until 30 days after [14]. Studies investigating topical, intramuscular or inhalational glucocorticoids were excluded. If glucocorticoids were administered as part of an anti-hormone therapy regime; as chemotherapy; or as premedication before chemotherapy, trials were excluded. So were trials including patients with hypothalamic pituitary adrenal (HPA) axis dysfunction or long-term glucocorticoid treatment close to the time of surgery. The exposure to perioperative glucocorticoids should be compared to control groups either receiving placebo; standard of care; no intervention; or another intervention.

The outcomes of interest evaluated the long-term safety and potential benefits of oral or intravenous perioperative glucocorticoids for cancer surgery. The primary outcome, recurrence, was a composite of local, regional, and distant recurrence. OS was defined as time from surgery to death of any cause; DFS, also called relapse-free survival or recurrence-free survival, was defined as the time from surgery until death of any cause or cancer recurrence; and CSS was defined as the time from surgery to death from the same cancer. A follow-up time of at least six months after surgery was required.

### 2.3. Search Strategy for Identification of Studies

Literature searches were conducted using the electronic bibliographic databases PubMed, Embase, The Cochrane Central Register of Controlled Trials, and Web of Science applying medical subject headings and text words. The search did not contain any filters, language- or time restraints. The search string was peer-reviewed by two information specialists from separate reference libraries not otherwise affiliated with the project (see Table S1 for search string).

### 2.4. Study Selection and Data Collection

Two review authors (E.R.H. and A.O.) screened title and abstract of all items yielded by the search. If items met the predetermined criteria or it was doubtful whether they did, the full text was obtained. Disagreements between the authors were settled by discussion and third-party arbitration (R.P.H. or I.G.). Reasons for exclusions during the full text screening were logged.

If possible, articles not in English or Danish were translated for eligibility evaluation. If this was not possible, they were added to a list of relevant foreign titles (see Table S2). The reference lists of eligible articles were scanned for other relevant studies (snowball search). The following trial registers were searched for trials: clinicaltrialsregister.eu, isrctn.com, and clinicaltrials.gov. If a trial was relevant, a search for the potential published articles was performed.

The online data management software, Covidence (Covidence; Melbourne, Vic, Australia; www.covidence.org), was used for screening of records. Data extraction was done using a premade extraction form, and a pilot test was performed. If time-to-event data were only reported graphically, the software WebPlotDigitizer was used to extract it (Ankit Rohatgi; San Francisco, CA, USA; www.automeris.io/WebPlotDigitizer/), and guidelines imposed by Tierney et al were followed [15]. Equipotent doses of dexamethasone were calculated using an online corticosteroid calculator (ClinCalc LLC; Chicago, IL, USA; clincalc.com/Corticosteroids).

If required data were not provided within an article, the study authors were contacted in order to obtain these. If only short-term outcomes were reported, study authors were contacted in attempt to obtain potential results for long-term outcomes. A minimum of two email attempts were made at least 14 days apart.

The following variables for eligible studies were extracted if reported: type of study; study design; trial size; type of cancer; Union for International Cancer Control (UICC) stage; type of surgery; type, dose and frequency of glucocorticoid administered; time to follow-up; reasons for loss of follow-up; causes of death; cancer recurrence; death; and time to event data (HR) (see Table 1 for study characteristics and Table 2 for patient characteristics).

### 2.5. Risk of Bias in Individual Studies

Risk of bias assessment for randomised studies was performed using the Cochrane Risk of Bias tool 1.0 (RoB 1.0) [16]. This covers assessment of six items, for which the risk of bias was judged either as high risk; low risk; or unclear risk.

Risk of bias for non-randomised studies was assessed using the Newcastle-Ottawa Scale (NOS) [17]. This covers three items, and studies were judged as having either low, medium or high risk of bias. The assessments were done at study level and blinded to the assessor (ERH).

### 2.6. Statistical Analyses

For each outcome, events and sample size for postoperative year one, three and five were extracted as well as HR and 95% confidence interval (CI). The extracted events were as follows: cancer recurrence for the primary outcome recurrence; death from any cause for OS; death of any cause or cancer recurrence, whichever came first, for DFS; and death from the same cancer for CSS.

Frequency meta-analyses of extracted event measures were performed by applying a random effect model (inverse variance) calculating risk ratios and 95% CI using the software tool RevMan 5.3 [18]. The DerSimonian-Laird (DL) tau^2^ estimator was applied and heterogeneity was assessed by Chi^2^ testing and I^2^ statistics.

Time-to-event meta-analyses of extracted hazard-ratios were performed by applying a random effect model (inverse variance) calculating HR using the statistics program R-studio and applying the packages meta, metagen and metafor. The Sidik-Jonkman estimator for tau^2^ was applied with Hartung-Knapp adjustment in the given random effect model [35]. Prediction intervals were calculated to provide estimates of the expected effect size of future studies based on current evidence [36,37]. Heterogeneity was assessed by Chi^2^ testing and I^2^ statistics.

Publication bias was assessed by Egger’s test [38], and funnel plots of unadjusted and adjusted OS hazard-ratios were created for visual inspection. Selective reporting bias was included in RoB 1.0, and it was assessed for non-randomised studies by comparing analyses specified in the method sections with analyses actually reported in the result sections.

All included studies were separated in two groups according to study design: non-randomised and randomised studies. Statistical analyses were done independently for each of the two groups.

The impact of the following factors on outcomes were pre-specified as subgroup analyses of interest: glucocorticoid type; timing in relation to surgery, frequency and dose of the glucocorticoid administration; cancer type; and time and magnitude of the surgery. It was also pre-specified that a fixed effect model would be applied on analyses were I^2^ was below 20%.

## 3. Results

### 3.1. Study Selection

**Figure 1:**
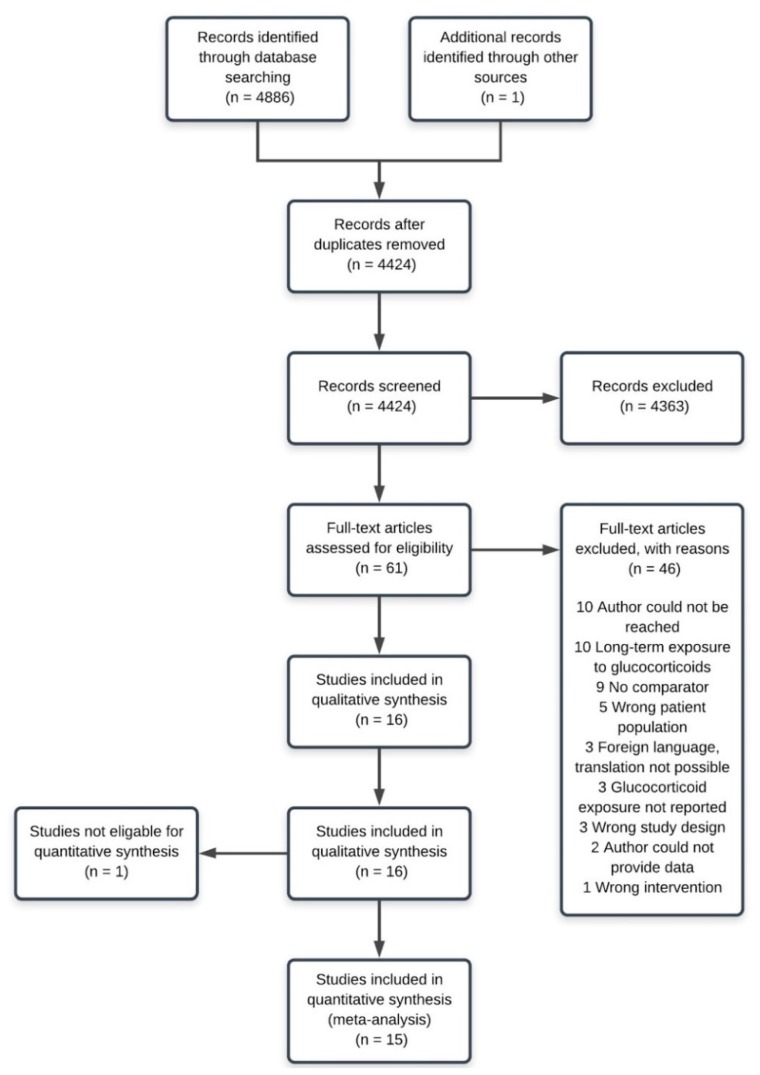
A flow diagram according to the Preferred Reporting Items for Systematic Reviews and Meta-Analyses (PRISMA) that maps the phases of the study selection process along with the number of records identified; excluded (and the reasons for this); and ultimately included in the systematic review and meta-analysis.

The search was run 27 March 2019 and yielded 4886 items. After duplicates were removed and screening was done, 16 studies were included in the systematic review. One of these could not be included in the meta-analysis as it did not provide any extractable data [22] (see Figure 1 for PRISMA flow diagram). A total of 25 authors were contacted in an attempt to gain additional information and one of them provided the requested data [32] (see Table S3 for contact attempts). One ongoing trial was found; completion expected in 2024 (clinicaltrials.gov ID: NCT04025840 [39]).

### 3.2. Study and Patient Characteristics

The included studies reported on a variety of eight cancer types: pancreatic adenocarcinoma [19,28], non-small-cell lung cancer [20,23,34], ovarian [21], rectal [22,33], colorectal [25,31], endometrial [26], oesophageal [27,29,30,32], and breast cancer [24]. Sample sizes range from 37 [27] to 2628 [24] patients and the follow-up periods from six months to ten years. The glucocorticoid exposures ranged from a single administration of low dose dexamethasone to repetitive very high doses of methylprednisolone. The timing of glucocorticoid administration varied from five days before and after surgery to a single administration just preoperatively. More than twice as many women (5244) as men (2407) were included. This difference was carried mainly by one study reporting on approximately 2600 women with breast cancer [24].

Six studies reported the primary outcome recurrence [21,22,24,25,26,32]. One of these was analysed as two separate studies, and will be referred to as two studies, because the participants were divided into two study populations according to lymph node dissemination [25]. Fifteen studies reported OS [19,20,22,23,24,25,26,27,28,29,30,31,32,33] and seven studies reported DFS [20,23,26,31,32,33,34]. Several studies reported recurrence-free survival or progression-free survival; however, this definition was in accordance with our predetermined definition of DFS and was analysed as such. One of the studies did not report any extractable data and was left out of the meta-analysis [22]. Only one study reported CSS [30], and a meta-analysis could not be performed for this outcome.

Four of the included studies were randomised controlled trials [22,29,31,32] (one of these was the study not included in the meta-analysis [22]) and the remaining twelve were retrospective cohort studies. Eleven studies found no association between the use of perioperative glucocorticoids and any of our outcomes [21,22,24,25,26,27,29,30,31,32]; four studies found associations in favour of glucocorticoids [19,23,28,34]; and two studies found associations in favour of the control group [20,33] (see Supplementary Material, Table S4 for all study results).

### 3.3. Risk of Bias within Studies

Not all risk of bias analyses could be done blinded: two Chinese articles [22,34] had to be unblinded in order to obtain translator assistance, and one article [31] was a follow-up analysis of a previously randomised controlled trial [40] and was unblinded in order to review the original description of the randomisation process.

Of the five studies eligible for the recurrence analyses, three had a low risk of bias [21,24,26]; two a medium risk of bias [25]; and one study was a randomised trial with almost entirely unclear risk of bias [32]. In the OS analyses, six non-randomised studies had a low risk of bias [19,20,23,24,26,33], and five had a medium risk [25,27,28,30]. Of the eligible randomised trials, one had an unclear risk of bias [32] and two had a low risk [29,31]. The DFS analyses included non-randomised studies with only low risks of bias [20,23,26,33,34]; one randomised study with an unclear risk of bias [32] and one with a low risk [31] (see Table 3 for risk of bias analyses).

### 3.4. Recurrence

Six studies provided eligible data for recurrence meta-analyses [21,24,25,26,32] yielding a total of 3586 patients of whom 576 (16%) received glucocorticoids and 3010 (84%) either had placebo or no treatment. After five years, 135 patients (30%) in the glucocorticoid group and 380 patients (14%) in the control group had developed recurrence.

No randomised studies reported recurrence, and the meta-analysis was therefore only done for non-randomised studies. The frequency meta-analysis of 1-year follow-up of the five studies showed no significant association between perioperative glucocorticoids and recurrence, RR 1.01, 95% CI (0.78–1.31), *p* = 0.93, with no heterogeneity, I^2^ = 0%. The 3-year follow-up also showed no association, RR = 1.00, 95% (0.85–1.18), *p* = 0.97, with no heterogeneity, I^2^ = 0%. The 5-year follow-up of four of the studies [21,24,26,32] also showed no association, RR 1.04, 95% CI (0.87–1.25), *p* = 0.67, with minor heterogeneity, I^2^ = 21%.

**Figure 2:**
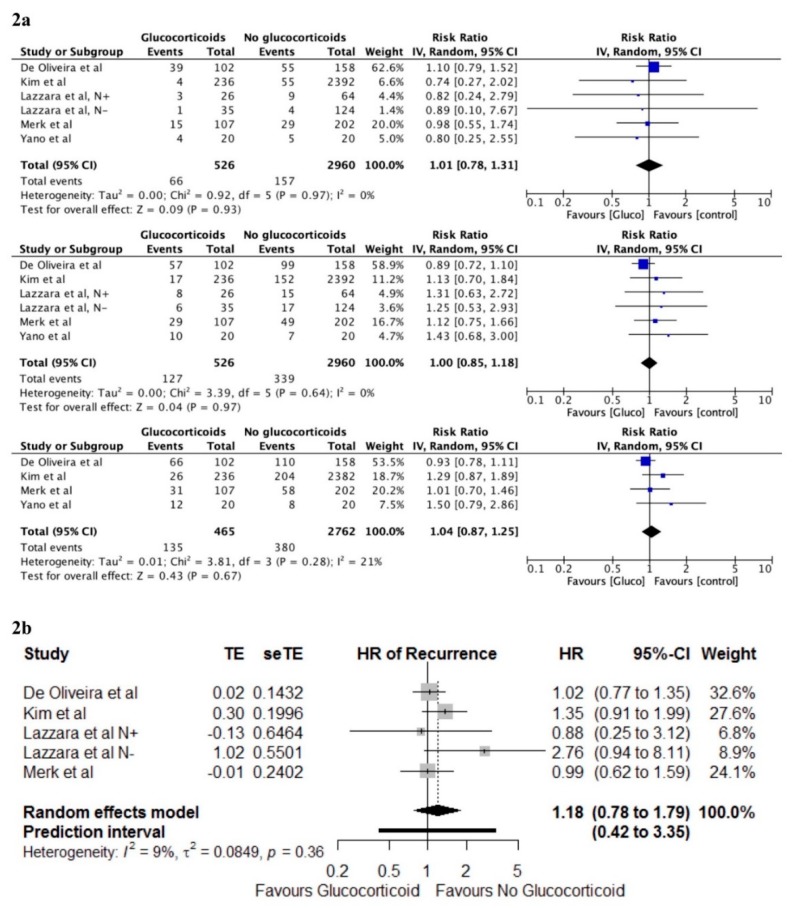
Forest plots of the effect sizes for recurrence after cancer surgery, random effect model. (**2a**) Frequency meta-analysis of non-randomised data from 0-1-, 0-3-, and 0-5-year follow-up (from top to bottom) (**2b**) Time-to-event meta-analysis of non-randomised data. CI = confidence interval; HR = hazard-ratio; TE = ln(HR); seTE = standard error for ln(HR).

The time-to-event meta-analysis of five [21,24,25,26] of the studies showed no group differences, HR 1.18, 95% CI (0.78–1.79), *p* = 0.32, with minor heterogeneity, I^2^ = 9%. Based on the current evidence, the prediction interval is 0.42 to 3.35 for possible future studies (see Figure 2 for recurrence forest plots).

### 3.5. Secondary Outcomes

#### Overall Survival

Fourteen studies provided eligible data for OS meta-analysis [19,20,23,24,25,26,27,28,29,30,31,32,33], yielding a total of 6806 patients of whom 1570 (23%) received glucocorticoids and 5236 (77%) either had placebo or no treatment. After five years, 428 patients (29%) in the glucocorticoid group and 1011 patients (20%) in the control group had died.

Out of studies eligible for data extraction, three randomised studies and ten non-randomised studies reported OS. The result of the frequency meta-analysis of 1-year follow-up for the randomised studies showed no association between perioperative glucocorticoids and OS, RR 1.20, 95% CI (0.55–2.60), *p* = 0.65, with no heterogeneity, I^2^ = 0%. The result of 3-year follow-up for the randomised trials were similar, RR 1.09, 95% CI (0.70–1.70), *p* = 0.69, with no heterogeneity, I^2^ = 0%. Data from the two randomised studies reporting 5-year follow-up resulted in an adverse estimated effect of glucocorticoids on OS, RR 1.64, 95% CI (1.00–2.71), *p* = 0.05, with no heterogeneity, I^2^ = 0%. Time-to-event meta-analysis of three randomised studies showed a HR of 1.46, 95% CI (0.61–3.46), *p* = 0.20, with no heterogeneity, I^2^ = 0%. Based on the current evidence, the prediction interval is 0.07 to 30.40 for future randomised studies.

The frequency meta-analysis of 1-year follow-up of the observational studies showed a significant association between perioperative glucocorticoids and increased OS, RR 0.70, 95% CI (0.51–0.97), *p* = 0.03, with low heterogeneity, I^2^ = 29%. Results after three years of follow-up were similar, RR = 0.89, 95% CI (0.71–1.13), *p* = 0.34, with substantial heterogeneity, I^2^ = 68%.

**Figure 3:**
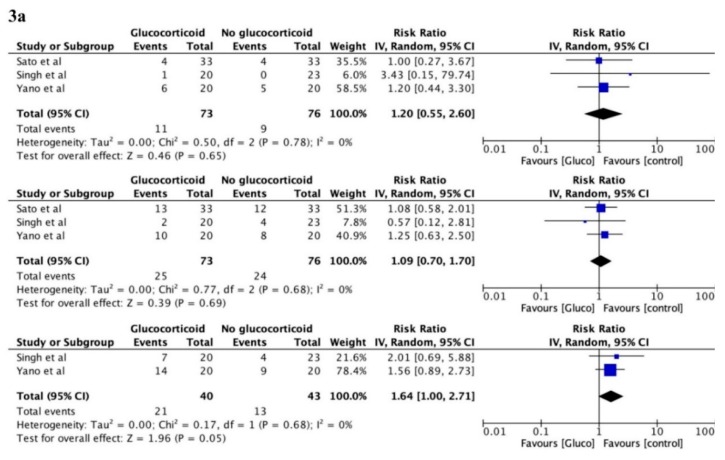

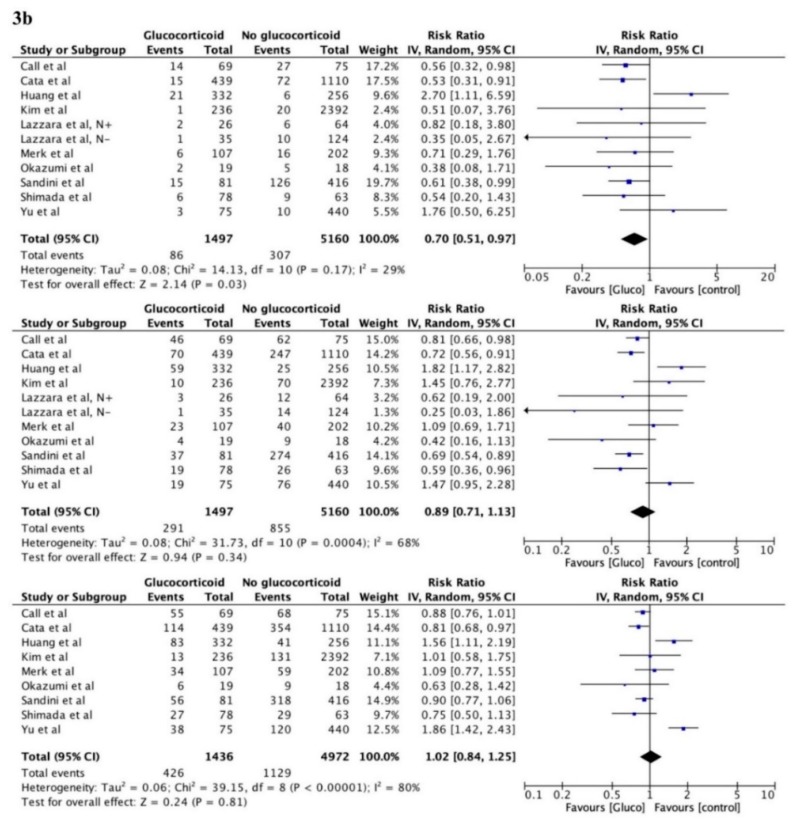

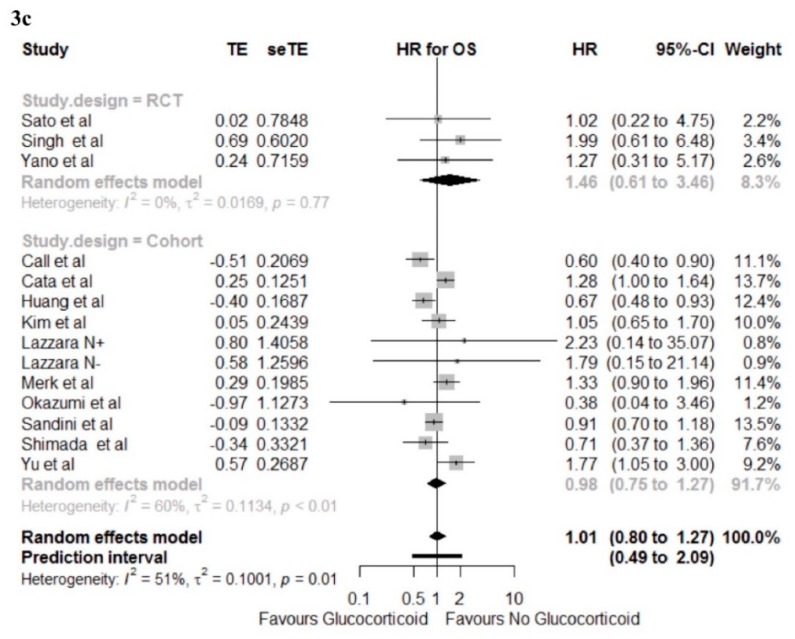
Forest plots of the effect sizes for overall survival after cancer surgery, random effect model. (**3a**) Frequency meta-analysis of randomised data from 0–1-, 0–3-, and 0–5-year follow-up (from top to bottom). **(3b**) Frequency meta-analysis of non-randomised data from 0–1-, 0–3-, and 0–5-year follow-up (from top to bottom). (**3c**) Time-to-event meta-analysis of randomised data (top) and non-randomised data (bottom). CI = confidence interval; HR = hazard-ratio; TE = ln(HR); seTE = standard error for ln(HR).

The 5-year follow-up of nine of the non-randomised studies [19,20,23,24,26,27,28,30,33] also showed no group differences, RR 1.02, 95% CI (0.84–1.25), *p* = 0.81, with a considerable heterogeneity, I^2^ = 80%. The time-to-event meta-analysis of the non-randomised studies also showed no association between OS and perioperative glucocorticoids, HR 0.98, 95% CI (0.75–1.27), *p* = 0.86, with moderate heterogeneity, I^2^ = 60%. Based on the current evidence, the prediction interval is 0.44 to 2.19 for possible future non-randomised studies (see Figure 3 for forest plots for OS).

### 3.6. Disease-Free Survival

Seven studies reported DFS [20,23,26,31,32,33,34], yielding a total of 3347 patients, of whom 1087 (32%) received glucocorticoids and 2260 (68%) either had placebo or no treatment. After five years, 214 patients (32%) in the glucocorticoid group and 587 patients (33%) in the control group had either died or developed recurrence.

The frequency meta-analysis of 1-year follow-up of the two randomised studies was not significant, RR 1.34, 95% CI (0.37–4.83), *p* = 0.65, with little heterogeneity, I^2^ = 14%. No association was found for the randomised studies three years after surgery either, RR 1.31, 95% CI (0.74–2.31), *p* = 0.35, with no heterogeneity, I^2^ = 0%. Similar results were found five years after surgery, RR 1.54, 95% CI (0.94–2.53), *p* = 0.09, with no heterogeneity, I^2^ = 0%.

**Figure 4:**
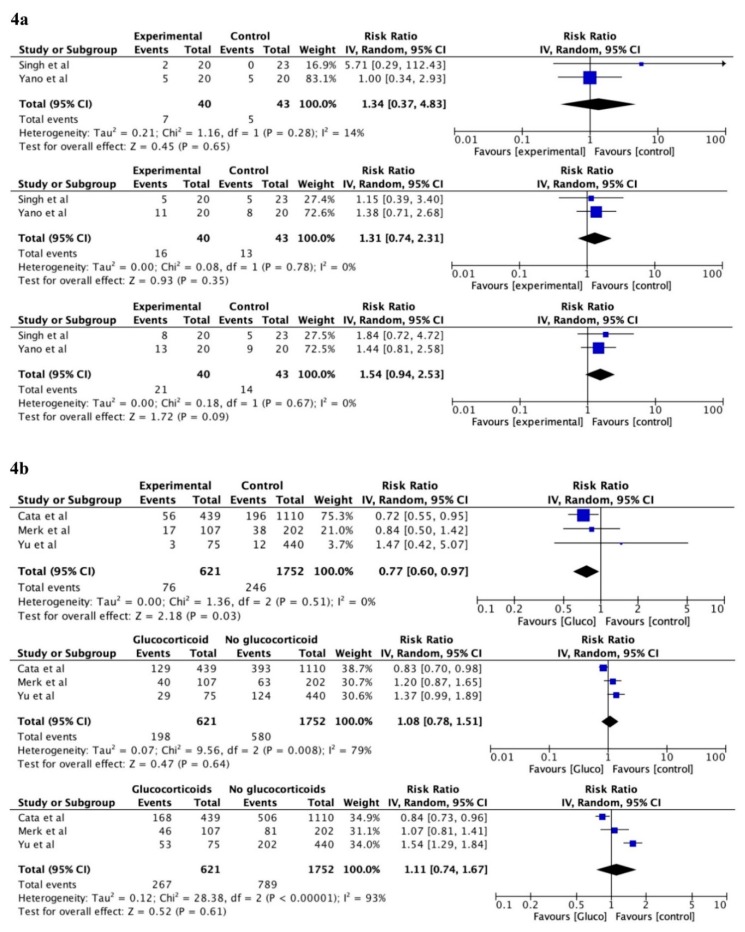

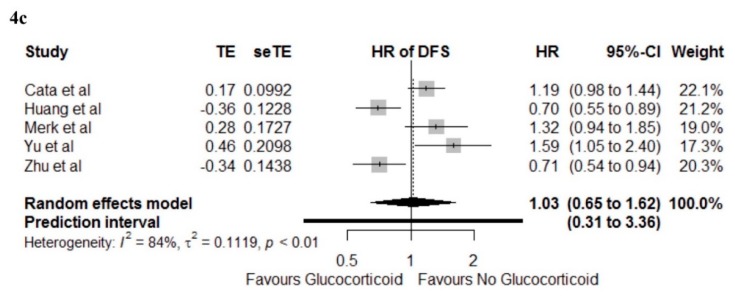
Forest plots of the effect sizes for disease-free survival after cancer surgery, random effect model. (**4a**) Frequency meta-analysis of randomised data from 0-1-, 0-3-, and 0-5-year follow-up (from top to bottom). (**4b**) Frequency meta-analysis of non-randomised data from 0-1-, 0-3-, and 0-5-year follow-up (from top to bottom). (**4c**) Time-to-event meta-analysis of non-randomised data. CI = confidence interval; HR = hazard-ratio; TE = ln(HR); seTE = standard error for ln(HR).

The result of 1-year follow-up of three of the observational studies [20,26,33] showed a significant association between perioperative glucocorticoids and increased DFS, RR 0.77, 95% CI (0.60–0.97), *p* = 0.03, with no heterogeneity, I^2^ = 0%. The 3-year follow-up of the non-randomised studies showed no association, RR = 1.08, 95% (0.78–1.51), *p* = 0.64, with substantial heterogeneity, I^2^ = 79%, and the 5-year follow-up results were similar, RR 1.11, 95% CI (0.74–1.67), *p* = 0.61, with considerable heterogeneity, I^2^ = 93%. The time-to-event meta-analysis of the five non-randomised studies [20,23,26,33,34] showed no group differences, HR 1.03, 95% CI (0.65–1.62), *p* = 0.88, with substantial heterogeneity, I^2^ = 84%. Based on the current evidence, the prediction interval is 0.31 to 3.36 for possible future non-randomised studies (see Figure 4 for forest plots for DFS).

### 3.7. Cancer Specific Survival

Only one study [30] reported this outcome, and therefore, a meta-analysis could not be performed. After five years, 19 patients (24%) in the study’s glucocorticoid group and 19 patients (30%) in the control group had died from their cancer. See Table 4 for results of all meta-analyses.

### 3.8. Subgroup Analyses

A subgroup analysis of the implication of cancer type on observational OS time-to-event data was performed (see Figure S1 for forest plot). The analysis was of OS, a secondary outcome, and not recurrence, as the number of recurrence reports was inadequate. The full subgroup analysis showed significant subgroup differences (*p* < 0.0001), meaning that glucocorticoids may possibly have different associations with different cancer types in terms of OS. Three observational studies [25,33] evaluated colorectal cancer and a significant association was found between the use of perioperative glucocorticoids and decreased OS, HR 1.78, 95% CI (1.57–2.03). The remaining cancer subgroups showed no within-group differences and generally included very few studies.

Furthermore, a subgroup analysis evaluating the association of accumulated equipotent dexamethasone dose with OS (20 mg dexamethasone as cut-off) was performed for observational studies. The test for subgroups differences was not statistically significant (*p* = 0.74); however, some confidence intervals were very wide and moderate heterogeneity was present, I^2^ = 60% (see Figure S2 for forest plot).

Additional subgroup analyses described in the method section were planned. However, considering the variability between studies, and because groupings within the analyses did not make practical sense, these analyses were not meaningful. For instance, most of the studies administrating a glucocorticoid type other than dexamethasone were also using larger doses than the dexamethasone trials and were of randomised study design. Additionally, all included studies evaluated major surgeries and a control group of smaller surgeries could not be formed for the surgical magnitude subgroup analysis. Due to the low number of studies included in analyses where I^2^ was below 20%, the pre-specified fixed effect models were not applied.

### 3.9. Sensitivity Analysis

A sensitivity analysis was performed by applying a random effects model on the adjusted OS data from observational studies if reported; however, only six studies reported adjusted HR. A random effect model showed no statistical group difference, HR 1.01, 95% CI (0.76–1.35), *p* = 0.92, with substantial heterogeneity, I^2^ = 69%. Based on the current evidence, the prediction interval is 0.42 to 2.43 for possible future studies. This effect estimate is comparable to the unadjusted results, and the width of the confidence intervals and prediction intervals are similar. This indicates that, in the available studies, adjustment for confounding does not seem to affect the results (see Figure S3 for forest plot for adjusted OS).

### 3.10. Risk of Bias across Studies

**Figure 5:**
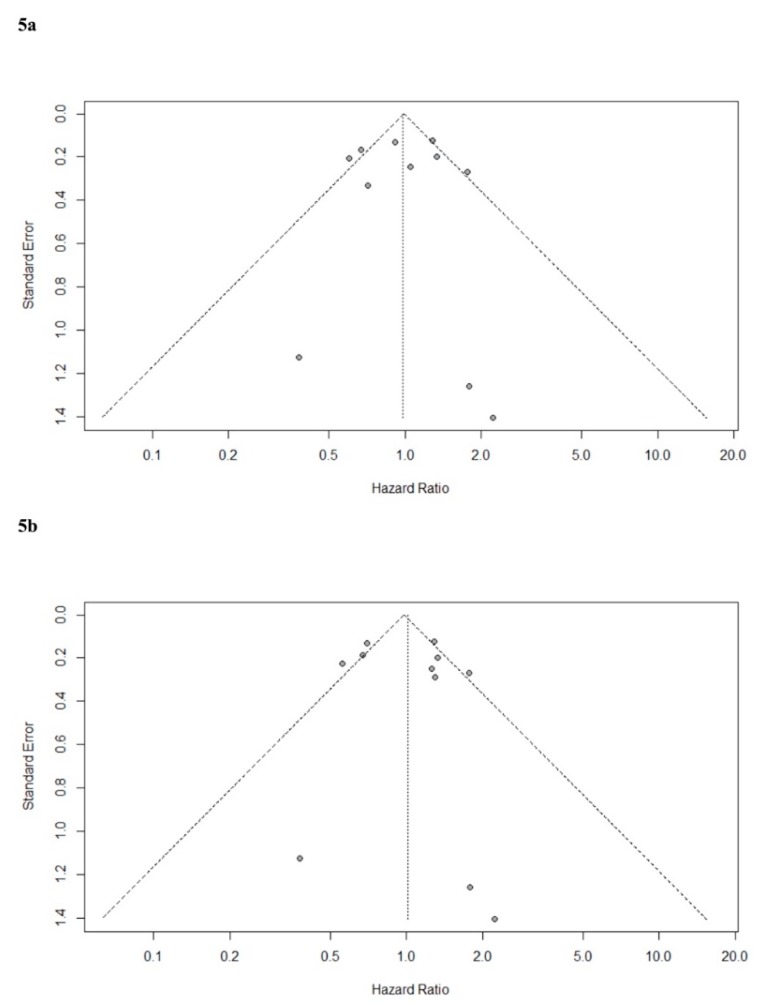
Funnel plots for visual assessment of publication biases. (**5a**) Unadjusted hazard-ratios for overall survival. (**5b**) Adjusted hazard-ratios for overall survival.

The funnel plot of the unadjusted HR for OS is in accordance with the adjusted version and shows a reasonable distribution. Eggers test concluded no evidence of publication bias (*p* = 0.90 and *p* = 0.70, respectively) (see Figure 5 for funnel plots).

## 4. Discussion

In the present study, no statistically significant association was found between the administration of perioperative glucocorticoids and cancer recurrence after surgery. Associations were found between perioperative glucocorticoids and increased 1-year OS and DFS after cancer surgery. An association was also found between glucocorticoids and decreased 5-year OS after cancer surgery. Furthermore, a subgroup analysis of the association between OS and different cancer types, suggested that patients undergoing colorectal cancer surgery had reduced survival when receiving glucocorticoids. The increased survival outcomes one year after surgery in the glucocorticoid group were most likely not carried by a reduction in metastatic disease since no association was found for 1-year recurrence and since 1-year DFS was increased. Part of the improved 1-year survival might be contributed to by the previously described reduced postoperative complication rates associated with perioperative glucocorticoids [41].

The result for 5-year OS suggesting an adverse effect of glucocorticoids was based solely on randomised data but from only two studies. The uncertainty in this result is reflected in the wide 95% confidence interval and in the very wide prediction interval for future randomised studies. The OS results based on non-randomised data also varied but generally trended towards no effect of glucocorticoids on OS over time. Prediction intervals were wide although not as much as for randomised studies. In summary, the current randomised and non-randomised data is too limited to draw final conclusions regarding the association between glucocorticoid administration and survival outcomes. The same point can be made for the primary outcome, recurrence, which was only represented in non-randomised studies and also showed a wide prediction interval in present meta-analysis.

The subgroup analysis of cancer types showed a negative association between glucocorticoids and OS only for patients having colorectal cancer surgery. Whether the colorectal cancer results are different by chance; due to low statistical power; or because of disease specific traits in patients with colorectal cancer is not known. However, future studies should investigate the possibility of cancer specific effect of glucocorticoids on long-term outcomes.

The predefined exploratory analyses regarding the impact of dose, administration frequency, timing and glucorticoid type on outcomes were not possible due to insufficient data. The fact that these intervention details are not being explored in the literature might lead to profound biological differences in intervention effects being overlooked. The pharmacological mechanism of action of glucocorticoids differs according to dose, but the clinical significance of this remains unclear [42]. It is plausible that low doses could have an anti-inflammatory effect (presumably beneficial), whereas high, repetitive doses might have immunosuppressive effect (presumably deleterious). Insufficient exploration of the effects of dosage, timing and frequency of administration are common even in randomised clinical trials. Therefore, future trials within this field should address these issues in the design phase to gain meaningful knowledge.

Even though extensive efforts were made to include all available evidence, this study is limited by several factors, such as the presence of clinical heterogeneity. The diversity of the eight different cancers should be considered, including the very different survival times between patients with pancreatic cancer and patients with breast cancer. Interstudy difference was also found in regard to study eligibility criteria; studies reporting recurrence data had little overlap in exclusion criteria, and their population demographics were heterogenous.

Two studies included a number of patients receiving other steroids than the study intervention. This could reduce an effect of the perioperative glucocorticoid intervention and thereby diminish possible associations with survival or recurrence. One of the studies had up to 5% of patients using chronic steroid in both groups [26]. This study was included, as the fraction was considered small. The other study had a larger fraction of patients receiving ‘postoperative dexamethasone’ in both groups [21] (75%), but it was not evident that this equalled long-term treatment, and the study was included. Despite being a randomised trial, one study had a significantly higher mean age in the glucocorticoid group compared to the control group (63.5 *±* 5.6 vs. 55.9 *±* 6.9) which could contribute to the negative (but statistically insignificant) association between glucocorticoids and survival concluded by the study [32].

Methodological diversity was present in the form of sample size variation and differences in follow up time. In spite of this, one large study did not carry most of the impact, according to forest plots. Statistical heterogeneity (I^2^) was either absent or very low (0% to 14%) for the analyses that only included randomised studies. In half of the analyses of non-randomised studies, I^2^ was 60% or more. However, for the significant results (1-year OS and 1-year DFS), I^2^ was low (29% and 0%, respectively). It would seem that randomised trials were more homogenous compared to non-randomised studies and more emphasis could be put on these results. However, the number of randomised trials were low, which could contribute to the low statistical heterogeneity among them.

Perioperative glucocorticoids are often administered as PONV prophylaxis. Risk factors for PONV are, among others: female gender; young age; and non-smoking status [43]. These are factors that are commonly known to favour survival in general. On the contrary, volatile anaesthetics and opioids are risk factors for PONV and are suspected of reducing survival and increasing cancer progression [44]. Furthermore, corticosteroids are administrated to treat adverse events in relation to blood transfusions, and meta-analytical evidence suggests that perioperative blood transfusions are associated with higher recurrence rates in patients undergoing colorectal cancer surgery [45]. Additionally, performing a more extensive surgical approach might lead the anaesthesiologist or surgeon to prescribe the patient (more) perioperative glucocorticoids for PONV prophylaxis, and the approach might also intensify the mechanisms that affect recurrence and survival. Hence, expanding the surgical approach (and thereby the inflammatory response) could be a confounding factor that affects the likelihood of receiving glucocorticoids and affects the long-term outcomes. Reasons for increasing surgical intensity could be but is not limited to the presence of a high tumour stage; acute indication of cancer surgery; or intraoperative complications.

Therefore, the risk of confounding by indication should be considered when investigating this topic and using data from observational studies. Based on the sensitivity analysis, confounding factors did not change the OS results, but this should be interpreted with care, as half of the studies in the sensitivity analysis did not report adjusted values [25,26,27,29,31,32,33], mainly because the included hazard-ratios were based on data extraction from Kaplan-Meier curves which results in unadjusted hazard-ratios. Conclusively, confounding factors may significantly affect the effect estimates of this meta-analysis and pose an important limitation of present paper.

This study cannot reject the hypothesis that perioperative glucocorticoids may have an impact on long-term survival outcomes after cancer surgery. The lack of randomised prospectively collected data and the extensive heterogeneity of the existing data weakens the conclusions of this review and warrants high quality data. It is therefore relevant to further investigate the impact of perioperative glucocorticoids. The biological plausibility of a causative relationship between glucocorticoids and long-term outcomes after cancer surgery should be evaluated in translational research focusing on both adaptive and innate immunological changes during the surgical stress response. In addition, trials such as the DREAMS trial [46] investigating the suspected positive effect of glucocorticoids on short-term outcomes should add secondary outcomes investigating long-term survival and recurrence. Additionally, national or cross-national large-scale register studies addressing this topic would be preferable, if they manage to adjust for confounding factors. In analyses of effects on oncological outcomes, it would be reasonable to exclude the immediate postoperative period where glucocorticoids are known to have an effect on survival that is not attributable to cancer recurrence.

## 5. Conclusions

The use of perioperative glucocorticoids was not found to be associated with recurrence after cancer surgery. We found associations between the use of perioperative glucocorticoids and increased OS and DFS one year after surgery as well as decreased OS five years after surgery. Furthermore, reduced OS was found for the subgroup of patients undergoing colorectal cancer surgery. But because the majority of our results are based on retrospective non-randomised data and is characterized by substantial clinical and statistical heterogeneity, no final conclusions regarding the long-term associations between perioperative glucocorticoids and OS or DFS can be made.

## Figures and Tables

**Table 1:** Study characteristics.

Study (Publication Year)	Study Type	Trial Recruitment Period	Inclusion Criteria (*n*)	Exclusion Criteria (*n*)	Exposure/Intervention (*n*)	Control Group (*n*)	Reported Outcome(s)
Call et al (2015) [19]	Retrospective cohort study	2001–2012	-Surgical resection of pancreatic adenocarcinoma stage I–IV	-Insufficient or illegible patient records (26)	Intraoperative dexamethasone treatment (1–10 mg) (69)	No intraoperative dexamethasone treatment (75)	OS
Cata et al (2016) [20]	Retrospective cohort study	2004–2014	-Surgery with curative intent of NSCLC stage I–III	-Palliative surgery -Secondary malignancy	Intraoperative dexamethasone treatment (4–20 mg) (439)	No intraoperative IV dexamethasone treatment (1110)	OS, DFS
De Oliveira et al (2014) [21]	Retrospective cohort study	1997–2007	-Optimal cytoreductive surgery of primary ovarian cancer stage I–IV	-Peritoneal tumour, benign or inconclusive pathology (45) -Tumour histology other than ovarian cancer (24) -Secondary surgical procedures (12) -Sub optimally debulked (5)	Intraoperative IV dexamethasone treatment (4–10 mg) (102)	No intraoperative IV dexamethasone treatment (158)	Recurrence
Gan et al (2015) [22]	Double-blinded randomized controlled study	2010–2011	-Intersphincteric resection; anterior resection or Miles resection of rectal cancer stage I–III -Rectal cancer verified with coloscopy -Consent to surgery	-Acute inflammatory or infectious disease -Bowel obstruction such as ileus -Bowel perforation or fever -Tumour recurrence or stage IV -No consent	Preoperative and postoperative Solu-Medrol treatment (0.4 mg/kg once daily from 5 d before surgery to 5 d after surgery) (50)	Placebo, administration identical with exposure regime (50)	
Huang et al (2018) [23]	Retrospective cohort study	2006–2009	-Lung resection or lobectomy of NSCLC -Above 18 years -NSCLC confirmed by postoperative pathology	-Primary malignant tumour in other place (15) -Recurrent metastatic lung tumour -Long-term steroid exposure -Impossible follow-up because of missing data (4)	Perioperative dexamethasone treatment (2–15 mg) (332)	No perioperative dexamethasone treatment (256)	OS, DFS
Kim et al (2019) [24]	Retrospective cohort study	2005–2010	-Breast conserving surgery or mastectomy for breast cancer stage I–III	-Multiple surgeries simultaneously (63) -Lack of anaesthetic or surgical information (21) - Steroid therapy for any reason (17)	Perioperative single dose of IV dexamethasone treatment (236)	No perioperative single dose of IV dexamethasone treatment (2392)	Recurrence, OS
Lazzara et al (2018) [25]	Retrospective cohort study	2012–2016	-Curative resection in adults with histologically proven stage I-III colorectal cancer	-Chronic inflammatory disease including bowel disease (IBD) -Long-term immunosuppressive therapy -Carcinoma in situ (intraepithelial or invasion of lamina propria) -Cancer infiltrating an adjacent organ or with radiological or surgical evidence of metastasis -Missing records of preoperative corticosteroids	Preoperative oral prednisone treatment (50 mg 13, 7 and 1 hour before surgery, totalling 150 mg) for all allergic patients (61)	No preoperative oral prednisone treatment (188)	OS, DFS
Merk et al (2016) [26]	Retrospective cohort study	2003–2007	-Surgery for endometrial cancer stage I–IV	-	Dexamethasone treatment (single dose 4–10 mg) (107)	No single dose dexamethasone treatment (202)	Recurrence, OS, DFS
Okazumi et al (2004) [27]	Retrospective cohort study	1995–1999	-Resection with three-field lymphadenectomy of the neck, mediastinum and abdomen of oesophageal squamous cell carcinoma stage 0–IVa	-Chemoradiotherapy before surgery (85)	Intraoperative methylprednisolone treatment (250 mg) and postoperative methylprednisolone treatment (125 mg) POD1 and POD2 (19)	No intraoperative methylprednisolone treatment (18)	OS
Sandini et al (2018) [28]	Retrospective cohort study	2007–2015	-Pancreaticoduodenectomy for pancreatic ductal adenocarcinoma	-Incomplete anaesthesia records (<2% of the cohort) -Invasive carcinoma from intraductal papillary mucinous neoplasms	Intraoperative dexamethasone (4–10 mg) (117)	No intraoperative dexamethasone (562)	OS
Sato et al (2002) [29]	Double blinded randomized controlled study	1996–1999	-Resection of oesophageal squamous cell carcinoma stage I–III	-Preoperative chemotherapy, radiation- or immunotherapy -Age over 76 years -Preoperative complications (such as liver cirrhosis, IDDM, CC < 60 mL/min, VC < 80%, FEV1 < 70%) -HBs-antigen or HCV-antibody positive -Multiple cancer -Old tuberculosis lesions	Preoperative methylprednisolone treatment (10 mg/kg body weight diluted in 100 mL physiologic saline within 30 minutes of the start of the surgery) (33)	A corresponding placebo infusion (33)	OS
Shimada et al (2004) [30]	Retrospective cohort study	1993–2000	-Radical esophagectomy for primary thoracic oesophageal squamous cell carcinoma stage I–IV	-Any preoperative adjuvant therapy	Intraoperative methylprednisolone treatment (250 mg) and postoperative methylprednisolone treatment (125 mg) POD1 and POD2 (78)	No intraoperative methylprednisolone treatment (63)	OS, CSS
Singh et al (2014) [31]	Follow up analysis of a previous double blinded randomized clinical trial	2006–2008	-Hemicolectomy for colon cancer stage I–III	-Immunosuppressive therapy including steroids -ASA score IV or V -Requirement of stoma -Unable to speak English -Significant cognitive impairment -Stage IV disease at time of surgery (3)	Preoperative dexamethasone treatment (8 mg at least 90 min before incision) (20)	Saline placebo at least 90 minutes before incision (23)	OS, DFS
Yano et al (2005) [32]	Double-blinded randomized controlled study	1997–1999	-Esophagectomy for thoracic oesophageal cancer	-	Preoperative methylprednisolone drip infusion (500 mg/body in saline 2 h preoperatively) (20)	Saline placebo 2 hours preoperatively (20)	Recurrence, OS, DFS
Yu et al (2015) [33]	Retrospective cohort study	2007–2011	-Curative resection of rectal cancer stage I–III -Histologically proven adenocarcinoma less than 15 cm from the anal verge	-Immunosuppressive therapy including recent steroid use (2) -Chronic inflammatory disease (including IBD) (5) -FAP diagnosis (2) -Multiple primary cancer (4) -Incomprehensive prescription records	Postoperative and/or intraoperative IV dexamethasone treatment (4–10 mg) (75)	No postoperative and/or intraoperative dexamethasone treatment (440)	OS, DFS
Zhu et al (2017) [34]	Retrospective cohort study	2003–2005	-Curative lung cancer surgery -No neoadjuvant therapy	-Other primary tumour(s) -Recurrence of previous lung cancer -Long-term steroid treatment -Missing records	Intraoperative dexamethasone treatment (94)	No intraoperative dexamethasone treatment (209)	DFS

Abbreviations: NSCLC = Non-small cell lung cancer; IBD = Inflammatory bowel disease; IDDM = Insulin dependent diabetes mellitus; CC = creatinine clearance; VC = vital capacity; FEV1 = forced expiratory volume in 1 second; FAP = Familial adenomatous polyposis.

**Table 2:** Patient characteristics.

Study (Publication Year)	*n*	Follow-Up, Years	Age		Gender M (%) F (%)	
			Glucocorticoid	Control	Glucocorticoid	Control
Call et al (2015) [19]	144	1.2^A^	65^A^	67 ^A^	33 (47.8) 36 (52.2)	43 (57.3) 32 (62.7)
Cata et al (2016) [20]	1549	1–12	65.4 ^C^	63.5 ^C^	176 (40.1) 263 (59.9)	603 (54.3) 507 (45.7)
De Oliveira et al (2014) [21]	260	4–10	57 ^A^	58 ^A^	0 (0.0) 102 (100)	0 (0.0) 158 (100)
Gan et al (2015) [22]	100	3.75 ^B^	53.2 ^B^	50.1 ^B^	24 (48) 26 (52)	28 (56) 22 (44)
Huang et al (2018) [23]	588	6–10	NA	NA	374 (63.6) * 214 (36.4) *	
Kim et al (2019) [24]	2628	5–10	49.5 ^B^	50.1 ^B^	0 (0.0) 236 (100)	0 (0.0) 2392 (100)
Lazzara et al (2018) [25]	249	0–4.75	66 ^A^	69 ^A^	26 (42.6) 35 (57.4)	110 (58.5) 78 (41.48)
Merk et al (2016) [26]	309	4.3 (G) ^A^ 3.9 (C) ^A^	64 ^A^	66 ^A^	0 (0.0) 107 (100)	0 (0.0) 202 (100)
Okazumi et al (2004) [27]	37	3–8	61 ^B^	62 ^B^	18 (94.7) 1 (5.3)	16 (88.9) 2 (0.1)
Sandini et al (2018) [28]	679	2 ^A^	65 ^A^	67 ^A^	38 (32.5) 79 (67.5)	284 (50.5) 278 (49.5)
Sato et al (2002) [29]	66	1.5–4.5	62 ^B^	64 ^B^	29 (88) 4 (12)	31 (94) 2 (6)
Shimada et al (2004) [30]	141	3-12	64 ^A^	64 ^A^	66 (84.6) 12 (15.4)	55 (87.3) 8 (12.7)
Singh et al (2014) [31]	43	4.8–6.5	72 ^A^	71 ^A^	6 (30.0) 14 (70.0)	13(56.5) 10(43.5)
Yano et al (2005) [32]	40	5	63.5 ^B^	55.9 ^B^	17 (85.0) 3 (15.0)	19 (95.0) 1 (5.0)
Yu et al (2015) [33]	515	3–7	60 ^A^	59 ^A^	47 (63) 28 (37)	250 (57) 190 (43)
Zhu et al (2017) [34]	303	2.5–8.45	61 ^A,^*		101 (33.3) * 202 (66.7) *	

^A^ Median, ^B^ Mean, * Numbers refer to overall population; Abbreviations: NA = not applicable; G = glucocorticoid group; C = control group.

**Table 3:** Risk of bias assessment of included studies, (**a**) the Newcastle-Ottawa Scale for non-randomised studies and (**b**) the Cochrane Risk of Bias evaluation 1.0 for randomised studies.

**a. The Newcastle-Ottawa Scale for Non-Randomised Studies**						
**Study (Publication Year)**	**Selection (Max 4 Stars)**	**Comparability (Max 2 Stars)**	**Outcome (Max 3 Stars)** **Loss to Follow up <10% Earns a Star if Unlikely to Introduce Bias**		**Total (Max 9 Stars)**	
Call et al (2015) [19]	***	**	**		7	
Cata et al (2016) [20]	***	**	**		7	
De Oliveira et al (2014) [21]	****	**	**		8	
Huang et al (2018) [23]	***	**	**		7	
Kim et al (2019) [24]	***	**	**		7	
Lazzara et al (2018) [25]	***		*		4	
Merk et al (2016) [26]	***	**	***		8	
Okazumi et al (2004) [27]	***		*		4	
Sandini et al (2018) [28]	***	*	**		6	
Shimada et al (2004) [30]	**	*	***		6	
Yu et al (2015) [33]	****	**	**		8	
Zhu et al (2017) [34]	***	**	**		7	
**b. The Cochrane Risk of Bias Evaluation 1.0 for Randomised Studies**						
**Study (Publication Year)**	**Selection Bias**	**Performance Bias**	**Detection Bias**	**Attrition Bias**	**Reporting Bias**	
	**Sequence Generation**	**Allocation Concealment**	**Blinding of participants and Personnel**	**Blinding of Outcome Assessors**	**Incomplete Outcome Data**	**Selective Outcome Reporting**
Yano et al (2005) [32]	Unclear risk of bias	Unclear risk of bias	Unclear risk of bias	Unclear risk of bias	Unclear risk of bias	Low risk of bias
Singh et al (2015) [31]	Low risk of bias	Low risk of bias	Low risk of bias	Unclear risk of bias	Low risk of bias	Low risk of bias
Sato et al (2002) [29]	Low risk of bias	Low risk of bias	Low risk of bias	Unclear risk of bias	Low risk of bias	Low risk of bias
Gan et al (2015) [22]	Low risk of bias	Low risk of bias	Low risk of bias	Low risk of bias	Low risk of bias	Low risk of bias

Each non-randomised study was judged according to a ‘star-system’ in which a number of stars (*) was awarded in line with predefined criteria by the Newcastle-Ottawa Scale. Three broad perspectives were judged, and zero to four stars could be given in the Selection category; zero to two stars in the Comparability category; and zero to three stars in the Outcome category. A total of zero to three stars to reflect a high risk of bias; four to six stars a medium risk of bias; and seven to nine stars a low risk of bias.

**Table 4:** Results of meta-analysis.

Outcome	Effect Estimate	95% CI	*p*-Value	Prediction Interval	I^2^
**Recurrence (non-randomised studies)**					
1-year (RR)	1.01	0.78–1.31	0.93	-	0%
3-year (RR)	1.00	0.85–1.18	0.97	-	0%
5-year (RR)	1.04	0.87–1.25	0.67	-	21%
Time-to-event (HR)	1.18	0.78–1.79	0.32	0.42–3.35	9%
**Overall survival (randomised studies)**					
1-year (RR)	1.20	0.55–2.60	0.65	-	0%
3-year (RR)	1.09	0,70–1.70	0.69	-	0%
5-year (RR)	1.64	1.00–2.71	0.05	-	0%
Time-to-event (HR)	1.46	0.61–3.46	0.20	0.07–30.4	0%
**Overall survival (non-randomised studies)**					
1-year (RR)	0.70 *	0.51–0.97	0.03	-	29%
3-year (RR)	0.89	0.71–1.13	0.34	-	68%
5-year (RR)	1.02	0.84–1.25	0.81	-	80%
Time-to-event (HR)	0.98	0.75–1.27	0.86	0.44–2.19	60%
**Disease-free survival (randomised studies)**					
1-year (RR)	1.34	0.37–4.83	0.65	-	14%
3-year (RR)	1.31	0.74–2.31	0.35	-	0%
5-year (RR)	1.54	0.94–2.53	0.09	-	0%
**Disease-free survival (non-randomised studies)**					
1-year (RR)	0.77 *	0.60–0.97	0.03	-	0%
3-year (RR)	1.08	0.78–1.51	0.64	-	79%
5-year (RR)	1.11	0.74–1.67	0.61	-	93%
Time-to-event (HR)	1.03	0.65–1.62	0.88	0.31–3.36	84%

Abbreviations: RR = risk ratio; HR = hazard ratio; CI = confidence interval. * Significant result.

## Supplementary Materials

The following are available online at https://www.mdpi.com/2072-6694/12/1/76/s1, Figure S1: Forest plot of the unadjusted hazard-ratios for overall survival after cancer surgery according to cancer type, applying a random effect model on time-to-event data, Figure S2: Forest plot of the unadjusted hazard-ratios for overall survival after cancer surgery according to dexamethasone dose (20 mg as cut-off), applying a random effect model on time-to-event data, Figure S3: Forest plot of the adjusted hazard-ratios for overall survival after cancer surgery, applying a random effect model on time-to-event data (sensitivity analysis), Table S1: PubMed search string for systematic review about perioperative glucocorticoid treatment for cancer surgery and long-term outcomes, date of search: 27 March 2019, Table S2: List of relevant foreign articles that could not be translated for eligibility evaluation for systematic review about perioperative glucocorticoid treatment for cancer surgery and long-term outcomes, Table S3: List of contact attempts for systematic review about perioperative glucocorticoid treatment for cancer surgery and long-term outcomes, Table S4: Results of individual studies included in systematic review about perioperative glucocorticoid treatment for cancer surgery and long-term outcomes for (**a**) recurrence, (**b**) unadjusted overall survival, (**c**) adjusted overall survival (**d**) disease-free survival and (**e**) cancer-specific survival.
