# Supplementary material for: Long-Term Outcomes after Use of Perioperative Glucocorticoids in Patients Undergoing Cancer Surgery: A Systematic Review and Meta-Analysis
Source: Cancers (Basel). 2019 Dec 27;12(1):76. doi: 10.3390/cancers12010076 (PMC7017046; doi:10.3390/cancers12010076)
Supplement: Supplementary file 1 [file cancers-12-00076-s001.pdf]

Supplementary Materials: Long-Term Outcomes after Use of Perioperative Glucocorticoids in Patients Undergoing Cancer Surgery: A Systematic Review and Meta-Analysis

Emma Rosenkrantz Hölmich, Rune Petring Hasselager, Michael Tvilling Madsen, Adile Orhan and Ismail Gögenur

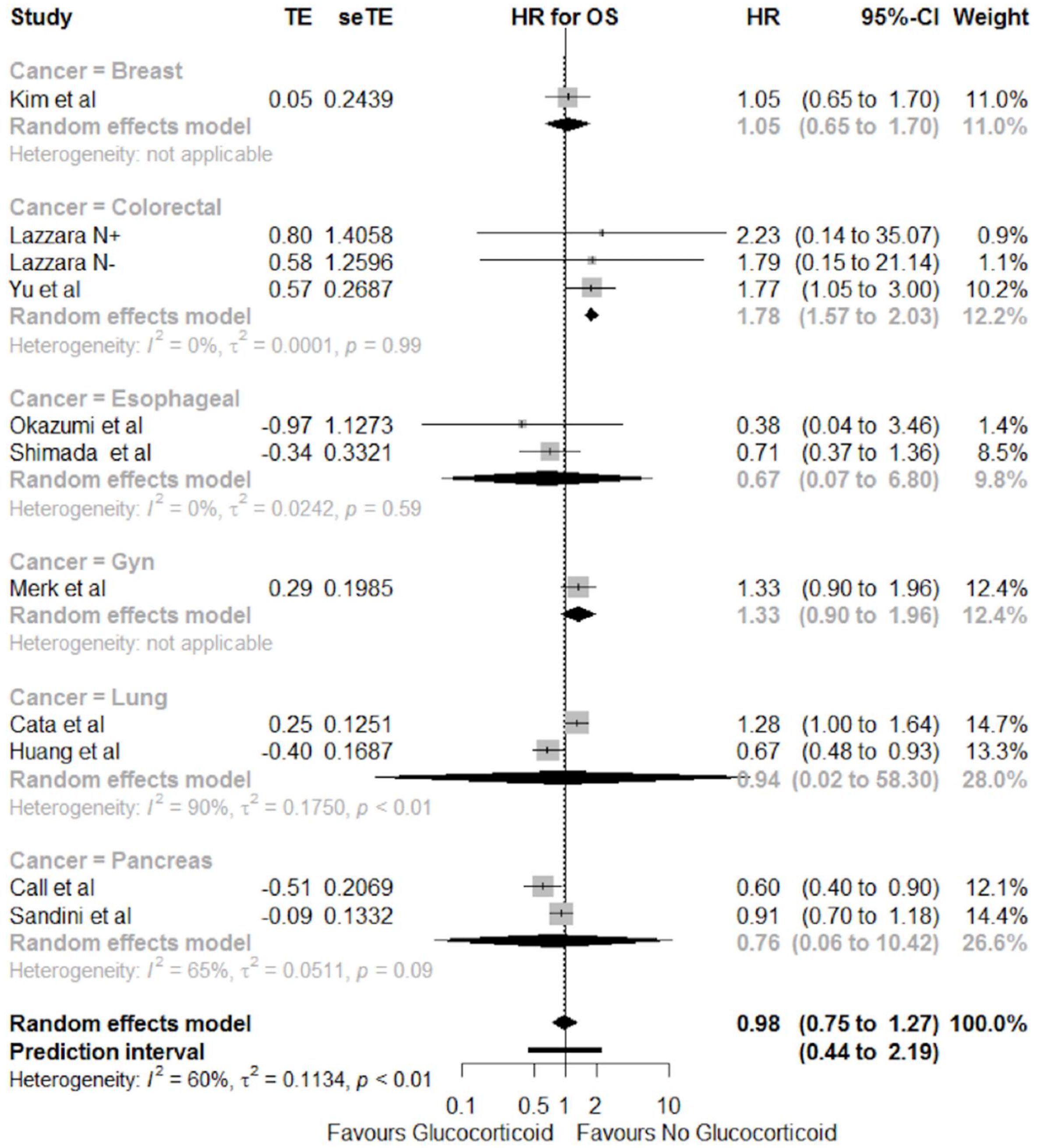

Figure S1. Forest plot of the unadjusted hazard-ratios for overall survival after cancer surgery according to cancer type, applying a random effect model on time-to-event data. CI = confidence interval; HR = hazard-ratio; OS = overall survival; TE = ln(HR); seTE = standard error for ln(HR).

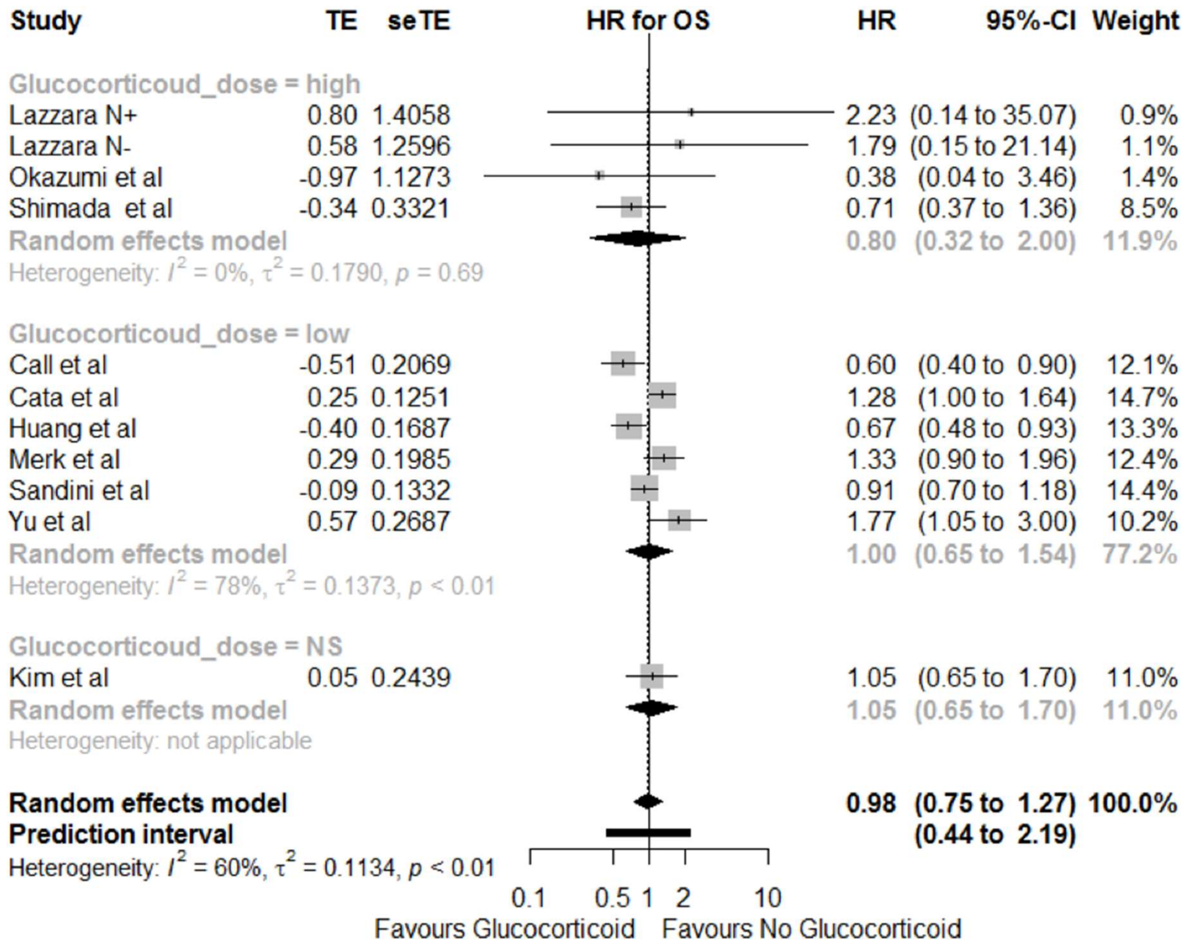

Figure S2. Forest plot of the unadjusted hazard-ratios for overall survival after cancer surgery according to dexamethasone dose (20 mg as cut-off), applying a random effect model on time-to-event data. CI = confidence interval; HR = hazard-ratio; OS = overall survival; TE = ln(HR); seTE = standard error for ln(HR).

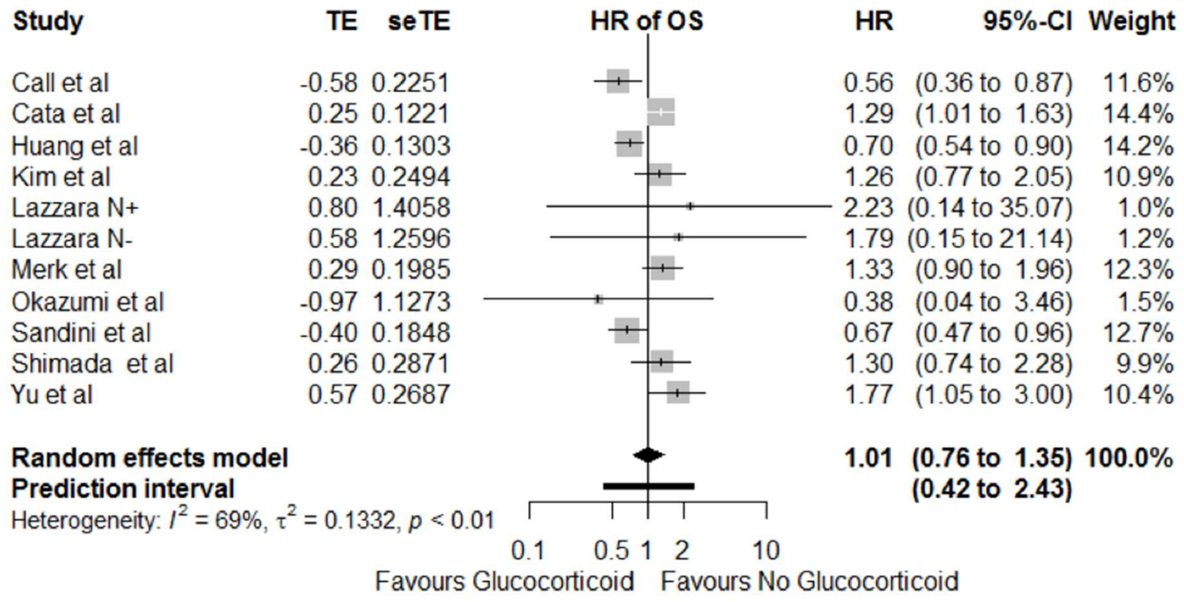

Figure S3. Forest plot of the adjusted hazard-ratios for overall survival after cancer surgery, applying a random effect model on time-to-event data (sensitivity analysis). CI = confidence interval; HR = hazard-ratio; OS = overall survival; TE = ln(HR); seTE = standard error for ln(HR).

Table S1. PubMed search string for systematic review about perioperative glucocorticoid treatment for cancer surgery and long-term outcomes, date of search: 27 March 2019.

(((((((("Neoplasms"[Mesh]) OR "Surgical Oncology"[Mesh])) OR (((((((((((((((((neoplasm) OR neoplasms) OR tumor) OR tumors) OR malignant) OR malignancy) OR malignancies) OR cancer) OR cancers) OR carcinoma) OR carcinomas) OR sarcoma) OR sarcomas) OR germinoma) OR germinomas) OR "oncologic surgery") OR "oncologic surgeries"))))
AND
(((((((("General Surgery"[Mesh]) OR "Surgical Procedures, Operative"[Mesh])) OR (((((((((((((Surgery) OR Surgeries) OR "Surgical treatment") OR "Surgical treatments") OR "Surgical intervention") OR "Surgical interventions") OR "Surgical procedure") OR "Surgical procedures") OR Operation) OR Operations) OR "Operative treatment") OR "Operative treatments") OR "Operative procedure") OR "Operative procedures")))))))
AND
((((((((((((((((((((((((((((((((((Betamethasone) OR Corticoids) OR Corticosteroid) OR Corticosterone) OR Cortisone) OR Dexamethasone) OR Fluprednisolone) OR Glucocorticoid) OR Glucocorticosteroid) OR Glucosteroid) OR Hydrocortisone) OR methylprednisolone) OR Prednisolone) OR Prednisone) OR Triamcinolone) OR Decadron) OR Cortef) OR Solucortef) OR Solu-cortef) OR Solu cortef) OR Cortef) OR Solumedrol) OR Solu-medrol) OR "Solu medrol") OR Medrol)) OR (((((((((((((("Triamcinolone"[Mesh]) OR "Steroids"[Mesh]) OR "Prednisone"[Mesh]) OR "Prednisolone"[Mesh]) OR "Methylprednisolone"[Mesh]) OR "Hydrocortisone"[Mesh]) OR "Glucocorticoids"[Pharmacological Action]) OR "Glucocorticoids"[Mesh]) OR "Fluprednisolone"[Mesh]) OR "Dexamethasone"[Mesh]) OR "Cortisone"[Mesh]) OR "Corticosterone"[Mesh]) OR "Betamethasone"[Mesh]) OR "Adrenal Cortex Hormones"[Mesh]))))))))))

Table S2. List of relevant foreign articles that could not be translated for eligibility evaluation for systematic review about perioperative glucocorticoid treatment for cancer surgery and long-term outcomes.

Author (Publication Year)	Study Title	Language
Fukai et al (2002) [1]	Therapeutic options which potentially cure patients with thymoma	Japanese
Ohira et al (2015) [2]	Perioperative steroid administration for colorectal cancer with synchronous unresectable hepatic metastases	Japanese
Yano et al (2005) [3]	Prophylactic administration of steroid for interstitial pneumonia after pulmonary resection for lung cancer	Japanese
Takemura et al (1999) [4]	Influence of corticosteroid preoperative administration for surgical stress of the oesophageal cancer patients during peri- and post-operative periods	Japanese

Table S3. List of contact attempts for systematic review about perioperative glucocorticoid treatment for cancer surgery and long-term outcomes.

**Author**	**Study Title (Year)**	**Comment**
Abou Zeid et al (2002) [5]	Dolasetron decreases postoperative nausea and vomiting after breast surgery	Author contacted for potential long-term data but did not respond.
Alkhamis et al (2014) [6]	Postoperative immunosuppression markers and the occurrence of sepsis in patients with benign and malignant disease	Author contacted for potential long-term data but did not respond.
Bononi et al (2010) [7]	Incidence and circumstances of cervical hematoma complicating thyroidectomy and its relationship to postoperative vomiting	Author contacted for potential long-term data and responded but was unable to provide data.
Chirila et al (2008) [8]	Cortisone treatment in prevention of pharyngocutaneous fistula after total laryngectomy	Author contacted for potential long-term data but did not respond.
Clayburgh et al (2017) [9]	A randomized controlled trial of corticosteroids for pain after transoral robotic surgery	Author contacted for potential long-term data but did not respond.
De Oliveira et al (2015) [10]	Perioperative dexamethasone and the development of chronic postmastectomy pain a single-center observational cohort study	Author contacted for potential long-term data but did not respond.
De Oliveira et al (2013) [11]	Is dexamethasone associated with recurrence of ovarian cancer?	Included study. Author contacted for potential survival data but did not respond.
Gomez-Hernandez et al (2010) [10]	Preoperative dexamethasone reduces postoperative pain, nausea and vomiting following mastectomy for breast cancer	Author contacted for potential long-term data but did not respond.
Park et al (2012) [12]	Efficacy of intraoperative, single-bolus corticosteroid administration to prevent postoperative acute respiratory failure after oesophageal cancer surgery	Author contacted for potential long-term data, but email address could not be reached.
DREAMS Trial (2017) [13]	Dexamethasone versus standard treatment for postoperative nausea and vomiting in gastrointestinal surgery: randomised controlled trial (DREAMS trial)	Author contacted for potential long-term data and responded but was unable to provide data.
McSorley et al (2018) [14]	The impact of preoperative dexamethasone on long-term survival following surgery for colorectal cancer	Author contacted for full article, as only the abstract was published, but was unable to provide this.
Takeuchi et al (2010) [15]	Factors influencing the long-term survival in patients with esophageal cancer who underwent esophagectomy after chemoradiotherapy	Author contacted for potential long-term data but did not respond.
Zhai et al (2013) [16]	Prospective randomized study of glucocorticoids in the impact of the liver function and the prognosis after hepatectomy of hepatocellular carcinoma	Author contacted for full article, as only the abstract was published, but did not respond.
Kirdak et al (2008) [17]	Does single, low-dose preoperative dexamethasone improve outcomes after colorectal surgery based on an enhanced recovery protocol? Double-blind, randomized clinical trial	Author contacted for potential long-term data and responded but was unable to provide data.

Pulitanò et al (2007) [18]	Prospective randomized study of the benefits of preoperative corticosteroid administration on hepatic ischemia-reperfusion injury and cytokine response in patients undergoing hepatic resection	Author contacted for potential long-term data and responded but was unable to provide data.
Yamashita et al (2001) [19]	Effects of preoperative steroid administration on surgical stress in hepatic resection: prospective randomized trial	Author contacted for potential long-term data but did not respond.
Shimada et al (1996) [20]	The effect of a perioperative steroid pulse on surgical stress in hepatic resection	Author contacted for potential long-term data, but email address could not be reached.
Shimada et al (2000) [21]	Clinical benefits of steroid therapy on surgical stress in patients with esophageal cancer	Author contacted for potential long-term data but did not respond.
Laaninen et al (2016) [22]	Perioperative hydrocortisone reduces major complications after pancreaticoduodenectomy: a randomized controlled trial	Author contacted for potential long-term data and responded but was unable to provide data.
Bolac et al (2013) [23]	The impact of postoperative nausea and vomiting prophylaxis with dexamethasone on postoperative wound complications in patients undergoing laparotomy for endometrial cancer	Author contacted for potential long-term data and responded but was unable to provide data.
Yano et al (2005) [24]	Is preoperative methylprednisolone beneficial for patients undergoing esophagectomy?	Included study. Author contacted, as recurrence data was not shown in article, and was able to provide data.
Shimada et al (2004) [25]	Effect of steroid therapy on postoperative course and survival of patients with thoracic esophageal carcinoma	Included study. Author contacted, as recurrence data was not shown in article, but did not respond.
Okazumi et al (2004) [26]	Development of less invasive surgical procedures for thoracic esophageal cancer	Included study. Author contacted for additional analyses, but email address could not be reached.
Gan et al (2015) [27]	Perioperative immunomodulatory therapy does not decrease postoperative recurrence rate of rectal cancer	Included study. Author contacted for recurrence and survival data, but did not respond?
Takeda et al (2003) [28]	Preoperative administration of methylprednisolone attenuates cytokine-induced respiratory failure after esophageal resection	Author contacted for potential long-term data but did not respond?

Table S4. Results of individual studies included in systematic review about perioperative glucocorticoid treatment for cancer surgery and long-term outcomes for (**a**) recurrence, (**b**) unadjusted overall survival, (**c**) adjusted overall survival (**d**) disease-free survival and (**e**) cancer-specific survival.

a

Non-randomised studies:

Author (Publication Year)	*n*	Gluco (*n*)	1-Year Recurrence (*n*)		3-Year Recurrence (*n*)		5-Year Recurrence (*n*)		HR	95% CI	Data Source
			Gluco	Control	Gluco	Control	Gluco	Control			
De Oliveira et al (2014) [11]	260	102	39	55	57	99	66	110	1.02	0.77–1.35	Kaplan-Meier curve
Kim et al (2019) [29]	2628	236	4	55	17	152	26	204	1.348	0.91–1.99	Pre-propensity competing risk univariate analysis and Kaplan-Meier curve
Lazzara et al N+ (2018) [30]	90	26	3	9	8	15	-	-	0.88	0.25–3.15	Kaplan-Meier curve
Lazzara et al N- (2018) [30]	159	35	1	4	6	17	-	-	2.76	0.94–8.12	Kaplan-Meier curve
Merk et al (2016) [31]	309	107	15	29	29	49	31	58	0.99	0.62–1.59	Univariate analysis and Kaplan-Meier curve
Yano et al (2005) [24]	40	20	4	5	10	7	12	8	-	-	Raw data provided by author

Abbreviations: *n* = number of participants; Gluco = glucocorticoid group; Control = control group; HR = hazard ratio; CI = confidence interval.

Randomised controlled trials:

Author (Publication Year)	*n*	Gluco (*n*)	1-Year Recurrence (*n*)		3-Year Recurrence (*n*)		5-Year Recurrence (*n*)		HR	95% CI	Data Source
			Gluco	Control	Gluco	Control	Gluco	Control			
Gan et al (2015) [27]	100	50	-	-	-	-	-	-	-	-	-

Abbreviations: *n* = number of participants; Gluco = glucocorticoid group; Control = control group; HR = hazard ratio; CI = confidence interval.

b

Non-randomised studies:

Author (Publication Year)	*n*	Gluco (*n*)	1-Year Survival (*n*)		3-Year Survival (*n*)		5-Year Survival (*n*)		HR	95% CI	Data Source
			Gluco	Control	Gluco	Control	Gluco	Control			

Call et al (2015) [32]	144	69	14	27	46	62	55	68	0.60	0.40–0.90	Kaplan-Meier curve and Univariate analysis
Cata et al (2016) [33]	1549	439	15	72	70	247	114	354	1.284	1.01–1.63	Kaplan-Meier curve and pre-propensity multivariate analysis
Huang et al (2018) [34]	588	332	21	6	59	25	83	41	0.67	0.48–0.93	Multivariate CPH analysis and Kaplan-Meier curve
Kim et al (2019) [29]	2628	236	1	20	10	70	13	131	1.051	0.65–1.70	Multivariate analysis and Kaplan-Meier curve
Lazzara et al N+ (2018) [30]	90	26	2	6	3	12	-	-	2.23	0.14–34.63	Kaplan-Meier curve
Lazzara et al N- (2018) [30]	159	35	1	10	1	14	-	-	1.79	0.15–20.92	Kaplan-Meier curve
Merk et al (2016) [31]	309	107	6	16	23	40	34	59	1.33	0.90–1.96	Kaplan-Meier curve
Okazumi et al (2004) [26]	37	19	2	5	4	9	6	9	0.38	0.04–3.32	Kaplan-Meier curve and survival rate calculation
Sandini et al (2018) [35]	497	81	15	126	37	274	56	318	0.91	0.70–0.18	Pre-propensity cox regression analysis and Kaplan-Meier curve
Shimada et al (2004) [25]	141	78	6	9	19	26	27	29	0.71	0.37–1.36	Adjusted CPH model and Kaplan-Meier curve
Yu et al (2015) [36]	515	75	3	10	19	76	38	120	1.77	1.05–3.01	Univariate analysis and Kaplan-Meier curve

Abbreviations: *n* = number of participants; Gluco = glucocorticoid group; Control = control group; HR = hazard ratio; CI = confidence interval.

Randomised controlled trials:

**Author (Publication Year)**	***n***	**Gluco (*n*)**	**1-Year Survival (*n*)**		**3-Year Survival (*n*)**		**5-Year Survival (*n*)**		**HR**	**95% CI**	**Data Source**
			**Gluco**	**Control**	**Gluco**	**Control**	**Gluco**	**Control**			
Gan et al (2015) [27]	100	50	-	-	-	-	-	-	-	-	-
Sato et al (2002) [37]	66	33	4	4	13	12	-	-	1.02	0.22–4.77	Kaplan-Meier curve
Singh et al (2014) [38]	43	20	1	0	2	4	7	4	1.99	0.61–6.46	Kaplan-Meier curve

Yano et al (2005) [24]	40	20	6	5	10	8	14	9	1.27	0.31–5.13	Univariate analysis and Kaplan-Meier curve

Abbreviations: *n* = number of participants; Gluco = glucocorticoid group; Control = control group; HR = hazard ratio; CI = confidence interval.

c

Non-randomised studies:

Author (Publication Year)	*n*	Gluco (*n*)	1-Year Survival (*n*)		3-Year Survival (*n*)		5-Year Survival (*n*)		HR	95% CI	Data Source
			Gluco	Control	Gluco	Control	Gluco	Control			
Call et al (2015) [39]	144	69	14	27	46	62	55	68	0.56	0.36–0.87	Kaplan-Meier curve and Multivariate model 2
Cata et al (2016) [33]	1549	439	15	72	70	247	114	354	1.286	1.01–1.63	Kaplan-Meier curve and pre-propensity multivariate analysis
Huang et al (2018) [34]	588	332	21	6	59	25	83	41	0.70	0.54–0.90	Multivariate CPH analysis and Kaplan-Meier curve
Kim et al (2019) [29]	2628	236	1	20	10	70	13	131	1.256	0.77–2.047	Multivariate analysis and Kaplan-Meier curve
Lazzara et al N+ (2018) [30]	90	26	2	6	3	12	-	-	2.23	0.14–34.63	Kaplan-Meier curve
Lazzara et al N- (2018) [30]	159	35	1	10	1	14	-	-	1.79	0.15–20.92	Kaplan-Meier curve

Merk et al (2016) [31]	309	107	6	16	23	40	34	59	1.33	0.90–1.96	Kaplan-Meier curve
Okazumi et al (2004) [26]	37	19	2	5	4	9	6	9	0.38	0.04–3.32	Kaplan-Meier curve and survival rate calculation
Sandini et al (2018) [35]	497	81	15	126	37	274	56	318	0.67	0.47–0.97	Pre-propensity cox regression analysis and Kaplan-Meier curve
Shimada et al (2004) [25]	141	78	6	9	19	26	27	29	1.30	0.74–2.28	Adjusted CPH model and Kaplan-Meier curve
Yu et al (2015) [36]	515	75	3	10	19	76	38	120	1.77	1.05–3.01	Univariate analysis and Kaplan-Meier curve

Abbreviations: *n* = number of participants; Gluco = glucocorticoid group; Control = control group; HR = hazard ratio; CI = confidence interval.

Randomised controlled trials

Author (Publication Year)	*n*	Gluco (*n*)	1-Year Survival (*n*)		3-Year Survival (*n*)		5-Year Survival (*n*)		HR	95% CI	Data Source
			Gluco	Control	Gluco	Control	Gluco	Control			
Gan et al (2015) [27]	100	50	-	-	-	-	-	-	-	-	-
Sato et al (2002) [37]	66	33	4	4	13	12	-	-	1.02	0.22–4.77	Kaplan-Meier curve
Singh et al (2014) [38]	43	20	1	0	2	4	7	4	1.99	0.61–6.46	Kaplan-Meier curve
Yano et al (2005) [24]	40	20	6	5	10	8	14	9	1.27	0.31–5.13	Univariate analysis and Kaplan-Meier curve

Abbreviations: *n* = number of participants; Gluco = glucocorticoid group; Control = control group; HR = hazard ratio; CI = confidence interval.

d

Retrospective cohort studies

Author (Publication Year)	*n*	Gluco (*n*)	1-Year Disease-Free Survival (*n*)		3-Year Disease-Free Survival (*n*)		5-Year Disease-Free Survival (*n*)		HR	95% CI	Data Source
			Gluco	Control	Gluco	Control	Gluco	Control			
Cata et al (2016) [33]	1549	439	56	196	129	393	168	506	1.185	0.98–1.44	Nonmatched univariate analysis and Kaplan-Meier curve
Huang et al (2018) [34]	588	332	-	-	-	-	-	-	0.70	0.55–0.89	Multivariate CPH model
Merk et al (2016) [31]	309	107	17	38	40	63	46	81	1.32	0.94–1.85	Kaplan-Meier curve
Yu et al (2015) [36]	515	75	3	12	29	124	53	202	1.59	1.05–2.39	Univariate analysis and Kaplan-Meier curve
Zhu et al (2017) [39]	303	94	-	-	-	-	-	-	0.712	0.55–0.97	Multivariate CPH model

Abbreviations: *n* = number of participants; Gluco = glucocorticoid group; Control = control group; HR = hazard ratio; CI = confidence interval.

Randomised controlled trials

Author (Publication Year)	*n*	Gluco (*n*)	1-Year Disease-Free Survival (*n*)		3-Year Disease-Free Survival (*n*)		5-Year Disease-Free Survival (*n*)		HR	95% CI	Data Source
			Gluco	Control	Gluco	Control	Gluco	Control			
Singh et al (2014) [38]	43	20	2	0	5	5	8	5	1.65	0.58–4.71	Kaplan-Meier curve and numbers at risk table
Yano et al (2005) [24]	40	20	5	5	11	8	13	9	-	-	Data provided by author

Abbreviations: *n* = number of participants; Gluco = glucocorticoid group; Control = control group; HR = hazard ratio; CI = confidence interval

e

Retrospective cohort studies

Author (Publication Year)	*n*	Gluco (*n*)	1-Year Cancer-Specific Survival (*n*)		3-Year Cancer-Specific Survival (*n*)		5-Year Cancer-Specific Survival (*n*)		HR	95% CI	Data Source
			Gluco	Control	Gluco	Control	Gluco	Control			
Shimada et al (2004) [25]	141	78	4	4	18	14	19	19	1.10	0.54–2.27	Kaplan-Meier curve

Abbreviations: *n* = number of participants; Gluco = glucocorticoid group; Control = control group; HR = hazard ratio; CI = confidence interval.

References

1. Fukai, I.; Yamakawa, Y.; Kiriyama, M.; Kaji, M.; Yano, T.; Sasaki, H.; Konishi, A.; Yukiue, H.; Fujii, Y. Therapeutic options which potentially cure patients with thymoma. *Kyobu Geka* **2002**, *55*, 959–964.
2. Ohira, G.; Miyauchi, H.; Narushima, K.; Kagaya, A.; Muto, Y.; Saito, H.; Matsubara, H. Perioperative Steroid Administration for Colorectal Cancer with Synchronous Unresectable Hepatic Metastases. *Gan to Kagaku Ryoho* **2015**, *42*, 1515–1517.
3. Yano, T.; Koga, T. Prophylactic administration of steroid for interstitial pneumonia after pulmonary resection for lung cancer. *Kyobu Geka* **2005**, *58*, 37–40.
4. Takemura, S.; Miya, K.; Takao, H.; Umemoto, T.; Saji, S. Influence of corticosteriod preoperative administration for surgical stress of the esophageal cancer patients during peri-and post-operative periods. *Jpn. J. Gastroenterol. Surg.* **1999**, *32*, 1133–1141, doi:10.5833/jjgs.32.1133.
5. Abou Zeid, H.; Al-Gahamdi, A.; Abdul-Hadi, M. Dolasetron decreases postoperative nausea and vomiting after breast surgery. *Breast J. Anaesth.* ***2002***, *8*, 216–221, doi:10.1046/j.1524-4741.2002.08405.x.
6. Alkhamis, T.; Ivic, D.; Wagner, J.; Ivic, J.; Dobrosevic, B.; Turina, I.; Kralik, K.; Barbic, J. Postoperative immunosuppression markers and the occurrence of sepsis in patients with benign and malignant disease. *Wien. Klin. Wochenschr.* **2014**, *126*, 774–784, doi:10.1007/s00508-014-0613-6.
7. Bononi, M.; Amore Bonapasta, S.; Vari, A.; Scarpini, M.; De Cesare, A.; Miccini, M.; Meucci, M.; Tocchi, A. Incidence and circumstances of cervical hematoma complicating thyroidectomy and its relationship to postoperative vomiting. *Head Neck* **2010**, *32*, 1173–1177, doi:10.1002/hed.21313.
8. Chirilă, M.; Bolboacă, S.; Tomescu, E.; Revesz, A.; Matioc, A.; Stamate, M.; Frandeş, I. Cortisone treatment in prevention of pharyngocutaneous fistula after total laryngectomy. *Chirurgia* **2008**, *103*, 553–557.
9. Clayburgh, D.; Stott, W.; Bolognone, R.; Palmer, A.; Achim, V.; Troob, S.; Li, R.; Brickman, D.; Graville, D.; Andersen, P.; et al. A randomized controlled trial of corticosteroids for pain after transoral robotic surgery. *Laryngoscope* **2017**, *127*, 2558–2564, doi:10.1002/lary.26625.
10. De Oliveira, G.S.; Bialek, J.M.; Turan, A.; McCarthy, R.J.; Sessler, D.I. Perioperative dexamethasone and the development of chronic postmastectomy pain a single-center observational cohort study. *Reg. Anesth. Pain Med.* **2015**, *40*, 539–544, doi:10.1097/aap.0000000000000301.
11. De Oliveira, G.S., Jr.; McCarthy, R.; Turan, A.; Schink, J.C.; Fitzgerald, P.C.; Sessler, D.I. Is dexamethasone associated with recurrence of ovarian cancer? *Anesth. Analg.* **2014**, *118*, 1213–1218, doi:10.1213/ANE.0b013e3182a5d656.
12. Park, S.Y.; Lee, H.S.; Jang, H.J.; Joo, J.; Zo, J.I. Efficacy of intraoperative, single-bolus corticosteroid administration to prevent postoperative acute respiratory failure after oesophageal cancer surgery. *Interact. Cardiovasc. Thorac. Surg.* **2012**, *15*, 639–643, doi:10.1093/icvts/ivs167.
13. DREAMS Trial Collaborators and West Midlands Research Collaborative. Dexamethasone versus standard treatment for postoperative nausea and vomiting in gastrointestinal surgery: Randomised controlled trial (DREAMS Trial). *BMJ* **2017**, *357*, j1455, doi:10.1136/bmj.j1455.
14. McSorley, S.; Khor, B.Y.; Roxburgh, C.; Horgan, P.; McMillan, D. The impact of preoperative dexamethasone on long-term survival following surgery for colorectal cancer. *J. Clin. Oncol.* **2018**, *36*, doi:10.1200/JCO.2018.36.4_suppl.644.
15. Takeuchi, H.; Saikawa, Y.; Oyama, T.; Ozawa, S.; Suda, K.; Wada, N.; Takahashi, T.; Nakamura, R.; Shigematsu, N.; Ando, N.; et al. Factors influencing the long-term survival in patients with esophageal cancer who underwent esophagectomy after chemoradiotherapy. *World J. Surg.* **2010**, *34*, 277–284, doi:10.1007/s00268-009-0331-9.
16. Zhai, J.; Cai, X.; Qu, S.P.; Zhang, Y.J.; Chen, X.T.; Wu, D. Prospective randomized study of glucocorticoids in the impact of the liver function and the prognosis after hepatectomy of hepatocellular carcinoma. *HPB* **2013**, *15*, 231, doi:10.1111/hpb.12093.
17. Kirdak, T.; Yilmazlar, A.; Cavun, S.; Ercan, I.; Yilmazlar, T. Does single, low-dose preoperative dexamethasone improve outcomes after colorectal surgery based on an enhanced recovery protocol? Double-blind, randomized clinical trial. *Am. Surg.* **2008**, *74*, 160–167.

18. Pulitanò, C.; Aldrighetti, L.; Arru, M.; Finazzi, R.; Soldini, L.; Catena, M.; Ferla, G. Prospective randomized study of the benefits of preoperative corticosteroid administration on hepatic ischemia-reperfusion injury and cytokine response in patients undergoing hepatic resection. *HPB* **2007**, *9*, 183–189, doi:10.1080/13651820701216984.
19. Yamashita, Y.; Shimada, M.; Hamatsu, T.; Rikimaru, T.; Tanaka, S.; Shirabe, K.; Sugimachi, K. Effects of preoperative steroid administration on surgical stress in hepatic resection: Prospective randomized trial. *Arch. Surg.* **2001**, *136*, 328–333, doi:10.1001/archsurg.136.3.328.
20. Shimada, M.; Saitoh, A.; Kano, T.; Takenaka, K.; Sugimachi, K. The effect of a perioperative steroid pulse on surgical stress in hepatic resection. *Int. Surg.* **1996**, *81*, 49–51.
21. Shimada, H.; Ochiai, T.; Okazumi, S.; Matsubara, H.; Nabeya, Y.; Miyazawa, Y.; Arima, M.; Funami, Y.; Hayashi, H.; Takeda, A.; et al. Clinical benefits of steroid therapy on surgical stress in patients with esophageal cancer. *Surgery* **2000**, *128*, 791–798, doi:10.1067/msy.2000.108614.
22. Laaninen, M.; Sand, J.; Nordback, I.; Vasama, K.; Laukkarinen, J. Perioperative hydrocortisone reduces major complications after pancreaticoduodenectomy: A randomized controlled trial. *Ann. Surg.* **2016**, *264*, 696–702, doi:10.1097/sla.0000000000001883.
23. Bolac, C.S.; Wallace, A.H.; Broadwater, G.; Havrilesky, L.J.; Habib, A.S. The impact of postoperative nausea and vomiting prophylaxis with dexamethasone on postoperative wound complications in patients undergoing laparotomy for endometrial cancer. *Anesth. Analg.* **2013**, *116*, 1041–1047, doi:10.1213/ANE.0b013e318276cf58.
24. Yano, M.; Taniguchi, M.; Tsujinaka, T.; Fujiwara, Y.; Yasuda, T.; Shiozaki, H.; Monden, M. Is preoperative methylprednisolone beneficial for patients undergoing esophagectomy? *Hepato Gastroenterol.* **2005**, *52*, 481–485. Retrieved from PubMed. (Accession No. 15816462).
25. Shimada, H.; Okazumi, S.; Matsubara, H.; Nabeya, Y.; Hayashi, H.; Shiratori, T.; Aoki, T.; Shuto, K.; Gunji, Y.; Ochiai, T. Effect of steroid therapy on postoperative course and survival of patients with thoracic esophageal carcinoma. *Esophagus* **2004**, *1*, 89–94, doi:10.1007/s10388-004-0014-4.
26. Okazumi, S.; Ochiai, T.; Shimada, H.; Matsubara, H.; Nabeya, Y.; Miyazawa, Y.; Shiratori, T.; Aoki, T.; Sugaya, M. Development of less invasive surgical procedures for thoracic esophageal cancer. *Dis. Esophagus* **2004**, *17*, 159–163, doi:10.1111/j.1442-2050.2004.00379.x.
27. Gan, Z.M.; Wang, X.D.; Lv, D.H.; Liu, D.; Li, L. Perioperative immunomodulatory therapy does not decrease postoperative recurrence rate of rectal cancer. *Nan Fang Yi Ke Da Xue Xue Bao* **2015**, *35*, 562–566, doi:10.3969/j.issn.1673-4254.2015.04.20.
28. Takeda, S.; Kim, C.; Ikezaki, H.; Nakanishi, K.; Sakamoto, A.; Okawa, K.; Miyashita, M.; Sasajima, K.; Tajiri, T.; Tanaka, K.; et al. Preoperative administration of methylprednisolone attenuates cytokine-induced respiratory failure after esophageal resection. *J. Nippon. Med. Sch.* **2003**, *70*, 16–20.
29. Kim, M.H.; Kim, D.W.; Park, S.; Kim, J.H.; Lee, K.Y.; Hwang, J.; Yoo, Y.C. Single dose of dexamethasone is not associated with postoperative recurrence and mortality in breast cancer patients: A propensity-matched cohort study. *BMC Cancer* **2019**, *19*, 251, doi:10.1186/s12885-019-5451-5.
30. Lazzara, C.; Cristina, D.; Iman, K.; Saverio, L.; Angela, A.; Giuseppe, N.; Giuseppe, C. Impact of preoperative corticosteroids on oncological outcomes following colorectal surgery for cancer. *Immunol. Endocr. Metab. Agents Med. Chem.* **2018**, *18*, 68–75, doi:10.2174/1871522218011809051 55822.
31. Merk, B.A.; Havrilesky, L.J.; Ehrisman, J.A.; Broadwater, G.; Habib, A.S. Impact of postoperative nausea and vomiting prophylaxis with dexamethasone on the risk of recurrence of endometrial cancer. *Curr. Med. Res. Opin.* **2016**, *32*, 453–458, doi:10.1185/03007995.2015.1123146.
32. Call, T.R.; Pace, N.L.; Thorup, D.B.; Maxfield, D.; Chortkoff, B.; Christensen, J.; Mulvihill, S.J. Factors associated with improved survival after resection of pancreatic adenocarcinoma: A multivariable model. *Anesthesiology* **2015**, *122*, 317–324, doi:10.1097/ALN.0000000000000489.
33. Cata, J.P.; Jones, J.; Sepesi, B.; Mehran, R.J.; Rodriguez-Restrepo, A.; Lasala, J.; Feng, L.; Gottumukkala, V. Lack of association between dexamethasone and long-term survival after non-small cell lung cancer surgery. *J. Cardiothorac. Vasc. Anesth.* **2016**, *30*, 930–935, doi:10.1053/j.jvca.2016.01.004.
34. Huang, W.W.; Zhu, W.Z.; Mu, D.L.; Ji, X.Q.; Nie, X.L.; Li, X.Y.; Wang, D.X.; Ma, D. Perioperative management may improve long-term survival in patients after lung cancer surgery: A retrospective cohort study. *Anesth. Analg.* **2018**, *126*, 1666–1674, doi:10.1213/ane.0000000000002886.

35. Sandini, M.; Ruscic, K.J.; Ferrone, C.R.; Warshaw, A.L.; Qadan, M.; Eikermann, M.; Lillemoe, K.D.; Fernandez-Del Castillo, C. Intraoperative dexamethasone decreases infectious complications after pancreaticoduodenectomy and is associated with long-term survival in pancreatic cancer. *Ann. Surg. Oncol.* **2018**, *25*, 4020–4026, doi:10.1245/s10434-018-6827-5.
36. Yu, H.C.; Luo, Y.X.; Peng, H.; Kang, L.; Huang, M.J.; Wang, J.P. Avoiding perioperative dexamethasone may improve the outcome of patients with rectal cancer. *Eur. J. Surg. Oncol.* **2015**, *41*, 667–673, doi:10.1016/j.ejso.2015.01.034.
37. Sato, N.; Koeda, K.; Ikeda, K.; Kimura, Y.; Aoki, K.; Iwaya, T.; Akiyama, Y.; Ishida, K.; Saito, K.; Endo, S. Randomized study of the benefits of preoperative corticosteroid administration on the postoperative morbidity and cytokine response in patients undergoing surgery for esophageal cancer. *Ann. Surg.* **2002**, *236*, 184–190, doi:10.1097/01.SLA.0000022025.67985.53.
38. Singh, P.P.; Lemanu, D.P.; Taylor, M.H.; Hill, A.G. Association between preoperative glucocorticoids and long-term survival and cancer recurrence after colectomy: Follow-up analysis of a previous randomized controlled trial. *Br. J. Anaesth.* **2014**, *113* (Suppl. 1), i68–i73, doi:10.1093/bja/aet577.
39. Zhu, W.Z.; Ji, X.Q.; Tan, H.Y. Retrospective cohort study of the association between perioperative factors and long-term survival after lung cancer operation. *Chin. J. Cancer Prev. Treat.* **2017**, *24*, 1387–1391, doi:10.16073/j.cnki.cjcpt.2017.19.011.

© 2020 by the authors. Licensee MDPI, Basel, Switzerland. This article is an open access article distributed under the terms and conditions of the Creative Commons Attribution (CC BY) license (http://creativecommons.org/licenses/by/4.0/).
